# *LincNEAT1* Encoded‐NEAT1‐31 Promotes Phagocytosis by Directly Activating the Aurora‐A–PI3K–AKT Pathway

**DOI:** 10.1002/advs.202413473

**Published:** 2025-05-08

**Authors:** Jie Li, Jing Zhang, Xixi Li, Xin Liu, Bo Zeng, Jiayue Luo, Huijin Wang, Hanbing Zhang, Xinya Gao

**Affiliations:** ^1^ Department of thyroid and breast surgery Guangzhou Women and Children's Medical Center Guangzhou Medical University Guangzhou 510623 P.R. China; ^2^ Institute of Reproductive Health and Perinatology Guangzhou Women and Children's Medical Center Guangzhou Medical University Guangzhou 510623 P.R. China; ^3^ Department of Neurosurgey The First Affiliated Hospital of Sun Yat‐sen University Guangzhou Guangdong 510080 P.R. China; ^4^ Department of Thoracic Surgery The First Affiliated Hospital Sun Yat‐sen University Guangzhou 510080 P.R. China; ^5^ Department of Neurosurgery Shanghai Deji Hospital Qingdao University Shanghai 200331 P.R. China

**Keywords:** Aurora‐A, LincNEAT1, macrophages, phagocytosis, protein kinase B

## Abstract

Macrophages play vital roles in innate and adaptive immunity, and their essential functions are mediated by phagocytosis and antigen presentation. Long non‐coding ribonucleic acid nuclear enriched abundant transcript 1 (*LincNEAT1*) is specifically translated during phagocytosis. Great efforts have been made to explore the potential mechanisms of action of these phagocytic checkpoints. However, the use of a systematic checkpoint scanning strategy has been far from satisfactory, and intrinsic phagocytic activators have not been fully elucidated. Therefore, in vitro phagocytosis assays are performed using primary healthy donor macrophages and breast cancer cells. An equal number of cells are subjected to ribosome profiling, and immune system‐specific phagocytic activators are identified. *LincNEAT1* and encoded micro peptide NEAT1‐31, are considerably upregulated in phagocytic macrophages. Moreover, purified NEAT1‐31 promoted the phagocytosis of multiple cancer cell‐types. Phosphoproteomic analysis reveals that NEAT1‐31 directly promoted Aurora‐A activity and activated phosphatidylinositol 3‐kinase/protein kinase B signaling. NEAT1‐31 enhanced the efficacy of anti‐CD47 in vivo and in vitro. Thus, the study identified a novel protein drug that can directly enhance the phagocytic function, thereby providing a new option for immunotherapy.

## Graphical abstract

*LincNEAT1* Encoded‐NEAT1‐31 micropeptide directly binds with Aurora‐A and enhanced AKT pathways to pormotes phagocytosis against multi cancer cells.

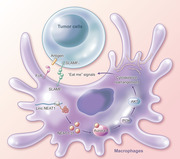

## 1 Introduction

Unprecedented success of immune checkpoint blockade therapy in suppressing cancer, by enhancing T‐cell responses, has been achieved. Nevertheless, drug resistance has prevented most patients from benefiting therefrom.^[^ ^1^, ^2^ ^]^ Advances in single‐cell multiomics have provided a deeper understanding of the complex interactions between various immune cell subpopulations and tumor cells in the tumor microenvironment, with results offering promising ideas for overcoming.^[^ ^3^, ^4^ ^]^ In addition to their essential role in innate immunity, macrophages imperatively participate in adaptive immunity by presenting antigens via phagocytosis. Regulated by receptor–ligand interactions between the target cell and phagocytic macrophage, termed “eat me” signals, this multistep cellular process involves target cell recognition, cellular engulfment, and lysosomal digestion.^[^ ^5^, ^6^ ^]^ Once the intrinsic “eat me” signals are activated by immunoreceptor tyrosine‐based activation motifs (ITAMs), the cytoskeletons of phagocytic macrophages are remodeled for phagocytosis. Conversely, cancer cells, such as cluster of differentiation (CD) 47, CD24, programmed death ligand (PDL)1, major histocompatibility complex‐I, stanniocalcin‐1, and disialoganglioside GD2 cells, evade immune clearance with the help of antiphagocytic molecules, termed “don't eat me” signals.^[^ ^7^, ^8^, ^9^, ^10^, ^11^, ^12^ ^]^ Most phagocytosis‐related ICB therapies focus on blocking “don't eat me” signals, such as those elicited by CD47 and PDL1 cells. However, few studies exist on phagocytosis activators.

In this study, we first established in vitro phagocytosis models using primary healthy donor macrophages and incubated them with the triple‐negative breast cancer (TNBC) cell line MDA‐MB‐231. Phagocytic macrophages were isolated and subjected to ribosome profiling. The lincRNA *LincNEAT1* was specifically translated during phagocytosis. *LincNEAT1* encodes the micro peptide NEAT1‐31. NEAT1‐31 promotes phagocytosis in vivo and in vitro. Proteomics and phosphoproteomics demonstrated that NEAT1‐31 activates Aurora A and the PI3K‐AKT pathway. Additionally, NEAT1‐31 enhanced the efficacy of anti‐CD47 therapy.

## 2 Results

### 2.1 Identification of Specific Phagocytic Macrophage Activators

**Figure 1:**
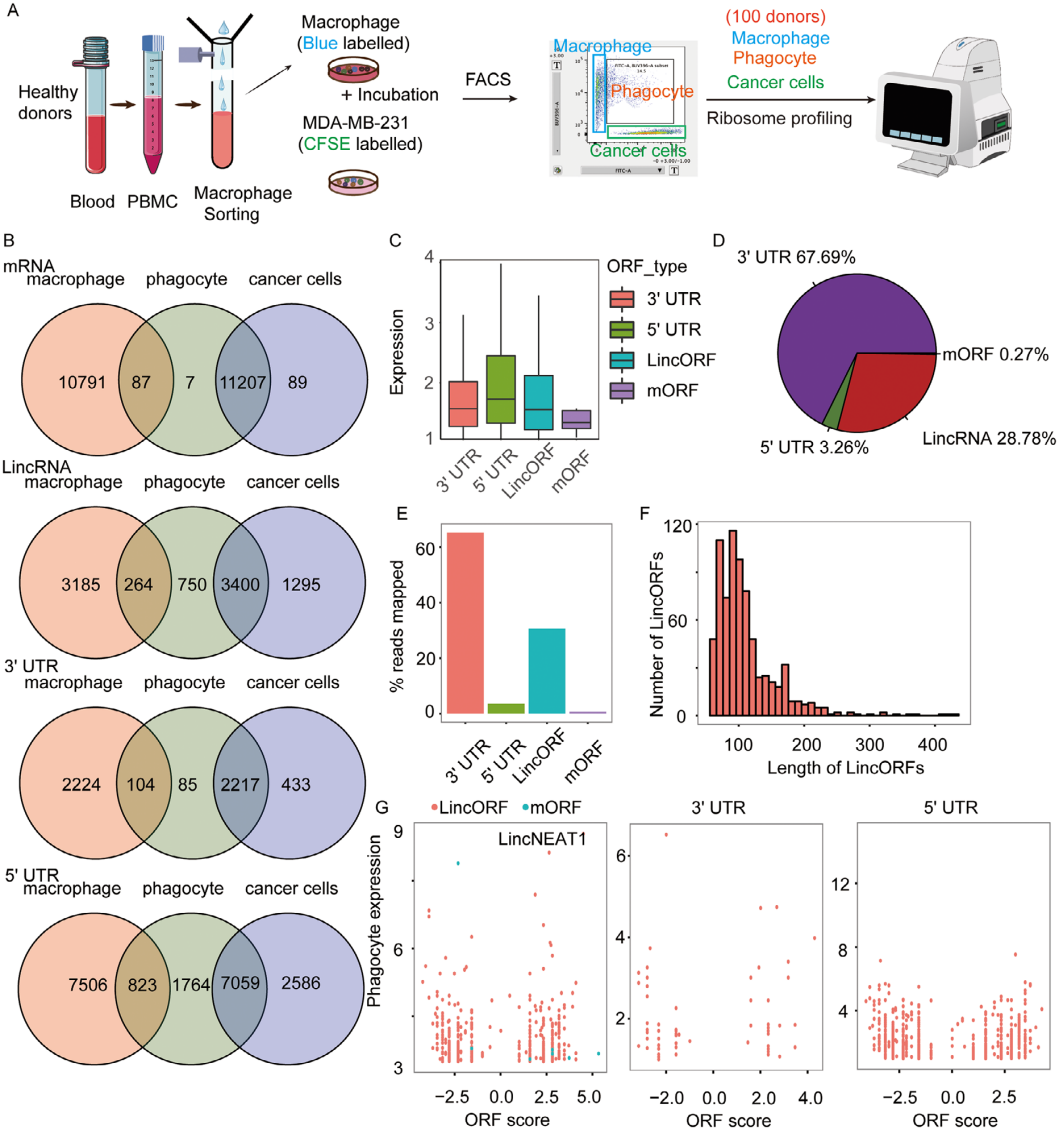
Ribosome profiling of phagocytic macrophages. A) Ribosome profiles of phagocytic macrophages, macrophages, and cancer cells are illustrated. Peripheral blood has been collected, PBMCs have been isolated, and macrophages have been induced and subsequently labeled with CellTrace Blue. Similarly, MDA‐MB‐231 breast cancer cells have been labeled with CFSE. Moreover, macrophages have been co‐cultured with MDA‐MB‐231 cells for 2 h and subjected to FACS analysis. Cells labeled with the blue cell tracker and CFSE are considered phagocytic macrophages. Cells labeled with only CFSE are MDA‐MB‐231 cells. Cells labeled with only CellTrace Blue are macrophages without phagocytic function. B) Venn plots of the dysregulated mRNA, *lincRNA*, 3′UTRs, and 5′UTRs are depicted. Phagocytic macrophage‐specific genes have been identified by comparing phagocytic macrophages with cancer cells (FPKM = 0 for MDA‐MB‐231 cells; FPKM > 0 for phagocytic macrophages). Dysregulated genes have been identified by comparing the expression levels of phagocytic macrophage‐specific genes with those of macrophages. C) Relative expression distributions of dysregulated mRNA, *lincRNA*, 3′UTRs, and 5′UTRs are shown. D) Pie diagrams of the distributions of dysregulated mRNA, *lincRNA*, 3′UTRs, and 5′UTRs are shown. E) RPFs are mapped according to the transcription and percentage of RPFs for each ORF. F) Distributions of the lengths of dysregulated *lincORFs* are depicted. G) Expression and ORF score of each dysregulated ORF and putatively translated lincRNA are ranked according to their expression in phagocytic macrophages. LncNEAT1, most abundantly expressed in phagocytic macrophages meeting the ORF score, is considerably upregulated in phagocytic macrophages along the X‐axis, ORF score, Y‐axis, and relative phagocytic macrophage expression. Abbreviations: PBMCs = peripheral blood mononuclear cells; CFSE = carboxyfluorescein diacetate N‐succinimidyl ester; FACS = fluorescence‐activated cell sorting; mRNA = messenger ribonucleic acid; *lincRNA* = long intergenic non‐coding RNA; UTRs = untranslated regions**;** FPKMs = fragments per kilobase per million mapped fragments; RPFs = ribosome protect fragments; ORF = open reading frame; mORF = mRNA ORF; *linc‐NEAT1* = long intergenic non‐coding RNA nuclear enriched abundant transcript 1.

To further elucidate the potential mechanisms of phagocytosis, identify and validate new immune checkpoints, search for targets that directly promote activity, and develop novel small molecule drugs, we first established an in vitro phagocytosis assay. Peripheral blood was collected. Thereafter, peripheral blood mononuclear cells (PBMCs) were isolated using the EasySep Direct Human Monocyte Isolation Kit (Stemcell Technologies, Vancouver, BC, Canada), macrophages were induced and cultured in ImmunoCult‐SF Macrophage Medium (Stemcell Technologies, Vancouver, BC, Canada), and labeled with CellTrace Blue (Thermo Fisher Scientific, cat# C34568). Similarly, MDA‐MB‐231 breast cancer cells were labeled with 5(6)‐carboxyfluorescein diacetate N‐succinimidyl ester (CFSE), co‐cultured with MDA‐MB‐231 cells for 2 h, and subjected to fluorescence‐activated cell sorting (FACS) analysis. Cells labeled with both blue cell tracker and CFSE simultaneously were considered as phagocytic macrophages. In addition to regular FACS, we used mild trypsinization to avoid cell adhesion and false‐positive results. Cells labeled with only the blue cell tracker were identified as macrophages without phagocytic ability. We isolated equal numbers of phagocytic macrophages, macrophages, and MDA‐MB‐231 and subjected to ribosome profiling (**Figure** **1**A). The ribosomal protection fragments (RPFs) were collected and subjected to transcriptional mapping. The reads mapped to the transcript regions showed a distinct 3‐nucleotide periodicity, suggesting reliable quality control (Figure S1A, Supporting Information). Principal component analysis was performed, and the RPF signatures differentiated cancer cells, phagocytic macrophages, and macrophages (Figure S1B, Supporting Information). For further confirmation, relative fragments per kilobase per million mapped fragments (FPKM) and RPF counts were used to analyze well‐studied phagocytosis checkpoints. The expressions of the “don't eat me” markers C‐type lectin‐like receptor 1 (CLEC‐1), sialic acid‐binding immunoglobulin‐like lectin 10 (Siglec‐10), signal regulatory protein alpha (SIRPα), leukocyte immunoglobulin‐like receptor subfamily B1 (LILRB1), and programmed death1 (PD1) were considerably higher in the macrophages than that in the phagocytic macrophages (Figure S1C, Supporting Information).

All the above data demonstrated reliable specificity and sensitivity. Thus, we applied individual analytical procedures. However, phagocytic macrophages actually involve two cell types‐ phagocytic macrophages “eating” cancer cells. To ensure the reliability of phagocytic macrophage activator identification, we applied a stricter analysis process. First, we identified phagocytic macrophage‐specific genes by comparing phagocytic macrophages with cancer cells (FPKM = 0 for MDA‐MB‐231 cells; FPKM > 0 for phagocytic macrophages). Second, we compared the expression of phagocytic macrophage‐specific genes in phagocytic macrophages with that in macrophages, ultimately identifying phagocytosis activators. Accordingly, Venn plots of the mRNAs, lncRNAs, 3′untranslated regions (UTRs), and 5′UTRs were constructed. A total of 2606 potential phagocytic macrophage activators were identified, including seven messenger RNA (mRNAs), 750 lincRNAs, 85 3′UTRs, and 1764 5′UTRs (Figure 1B; Tables S1–S4, Supporting Information). By contrast to conventional Venn plots, major overlaps of mRNAs (11 207), lincRNAs (3400), 3′UTRs (2217), and 5′UTRs (7059), were observed between phagocytic macrophages and MDA‐MB‐231 cells. This demonstrated the objectivity and reliability of the ribosome profiling method. Ribosome profiling of phagocytic macrophages included data from tumor cells and phagocytic macrophages, because “phagocytic macrophages” are phagocytic macrophages that eat MDA‐MB‐231 cells. This inevitably resulted in a substantial overlap between the two cells. The expression level of each group was summarized and revealed that the 5′UTRs exhibited the highest expression level (Figure 1C). Most RFPs were derived from 3′UTRs, which accounted for 67.69% of all RFPs (Figure 1D). Approximately 62.6% of the RFPs were mapped to 3′UTRs, 31.2% were mapped to long intergenic non‐coding RNAs (lincRNAs), and < 10% of the RFPs were mapped to mRNAs and 5′UTRs (Figure 1E). The distributions of long intergenic non‐coding open reading frames (lincORFs) and lincORF lengths were analyzed; and the number of lincORF peaking at 80–130 bp was determined (Figure 1F). The open reading frame (ORF) score was calculated to determine the extent of active translation, and the expression level was determined to validate the biological importance of each group. Putatively translated lincRNAs were ranked according to their expression in phagocytic macrophages. A putative lincRNA ORF (lincNEAT1‐31; ENST00000645023; ORF start at 2833 and end at 2928) with the highest abundance in phagocytic macrophages that met the ORF score was considerably upregulated (phagocytic macrophage FPKM = 11.62; cancer FPKM = 0; macrophage FPKM = 0.65; *P* = 3.01E‐06; fold change [FC] = 4.16) (Figure 1G).

### 2.2 Identification of NEAT1‐31 Encoded by *lncNEAT1*

**Figure 2:**
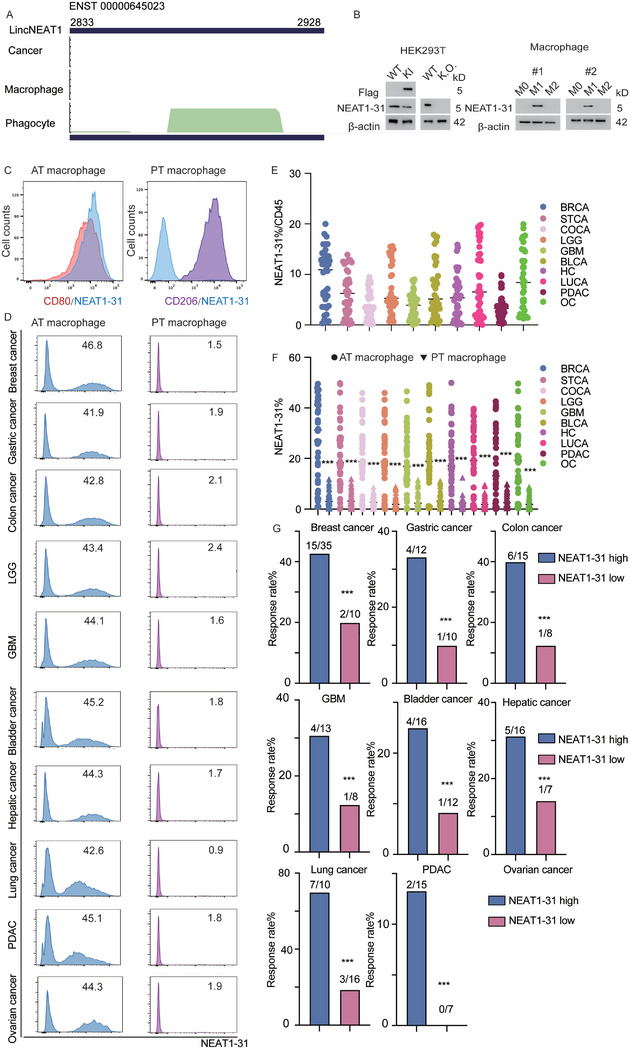
Anti‐tumor macrophages are enriched with *lincNEAT1*‐encoded NEAT‐31, better predicting the ICB therapy response. A) RPF density of *lincNEAT1* derived from ribosome profiling, NEAT1‐31 ORF, is located at 2833–2928 of ENST 00000645023, and the RPF reads are shown. B) Left: FLAG knock‐in and *lincNEAT1* knockout cell lines are established in HEK293 T‐cells, and IB has been performed to detect FLAG and NEAT1‐31. Right: THP1 and U‐937 cells have been induced into macrophages (M0/M1/M2), and NEAT1‐31 has been detected. C) FACS has been used to detect the distribution of NEAT1‐31 in anti/pro‐tumor macrophages. According to the expression of CD80 and CD206, NEAT1‐31 has been detected in each group. D) Macrophages have been derived from different sample specimens, and divided into anti/pro‐tumor macrophages, the expression of NEAT1‐31 has been detected. E) Percentages of NEAT1‐31^+^ macrophages derived from different cancer specimens are depicted (*n* = 40 for each cancer specimen). F) Percentages of NEAT1‐31^+^ macrophages in anti/pro‐tumor macrophages, derived from different cancer specimens, are shown, *n* = 40 for each cancer specimen; paired *T*‐test; *** *p* < 0.001. G) Ratios of NEAT1‐31^+^ macrophages to CD45 cells have been calculated using FACS. Patients have been divided into NEAT1‐31‐low and NEAT1‐31‐high groups, with the mean ratio of NEAT1‐31^+^ macrophages/CD45 cells serving as the cut‐off value. The RR of ICB therapy in each cohort was calculated and analyzed. The exact sample numbers were determined using the chi‐square test; *** *p* < 0.001. Abbreviations: *linc‐NEAT1*= long intergenic non‐coding acid (RNA) nuclear enriched abundant transcript 1; HEK = human embryonic kidney; WT = wild‐type; AT = antitumor; PT = protumor; BRCA = breast carcinoma; STCA = stomach carcinoma; COCA = colon carcinoma; LGG = low‐grade glioma; GBM = glioblastoma; BLCA = bladder urothelial carcinoma; HC = hepatocellular carcinoma; LUCA = lung carcinoma; PDAC = pancreatic ductal adenocarcinoma; OC = ovarian carcinoma; KI = knock in; K.O. =knock out; CD = cluster of differentiation; ICB = immune checkpoint blockade; RPF = ribosome protect fragment; ORF = open reading frame; THP1 = Tohoku Hospital Pediatrics‐1; FACS = fluorescence‐activated cell sorting; RR = response rate. All the experiments were repeated at least 3 times with similar results.

The *lincNEAT1* ORF was located in the chromosomal region 2833–2928 of the ENST 00000645023 transcript, and ribosome profiling analysis predicted that this ORF encoded for a 31‐amino acid micropeptide (MGIVGREWARCLYYMCDLKTLLGSELRLLNW, hereafter referred to as NEAT1‐31) (**Figure** **2**A). To confirm the translation of NEAT1‐31, we established a flag knock‐in and knock‐out system in human embryonic kidney (HEK) 293T cells; and Sanger sequencing was performed for confirmation (Figure S1D, Supporting Information). As no induction strategy for phagocytic macrophages was available, we treated macrophages with phorbol 12‐myristate 13‐acetate (PMA), lipopolysaccharide (LPS), or interleukin (IL) 4. Immunoblotting (IB) was performed to detect NEAT1‐31, using an anti‐FLAG antibody. Moreover, a specific monoclonal antibody was generated and applied to directly detect NEAT1‐31 (Figure 2B). The specificity of NEAT1‐31 was evaluated; chemosynthetic NEAT‐31 was applied as the blocking peptide; and was added in the dilution of NEAT1‐31 antibodies, to block the interaction of NEAT1‐31 and the antibody. For further confirmation, FACS was used to determine the proportion of NEAT1‐31^+^ cells among the different macrophages. Subsequently, we collected macrophages from healthy donors and probed them with antibodies against NEAT1‐31, CD80, and CD206. CD80^+^ macrophages were enriched with NEAT1‐31 and barely expressed in CD206^+^ macrophages. CD80 and CD206, two typical markers of antitumor/pro‐tumor (AT/PT) macrophages, were widely utilized^[^ ^13^, ^14^ ^]^ (Figure 2C). Subsequently, we collected different tumor samples and analyzed NEAT1‐31^+^ macrophages using FACS from 20 patients each. These tumor samples included breast carcinoma (BRCA), stomach carcinoma (STCA), colon carcinoma (COCA), low‐grade glioma (LGG), glioblastoma (GBM), bladder urothelial carcinoma (BLCA), hepatocellular carcinoma (HC), lung carcinoma (LUCA), pancreatic ductal adenocarcinoma (PDAC), and ovarian carcinoma (OC). BRCA had the highest proportion of NEAT1‐31^+^ macrophages, and the infiltration rates of NEAT1‐31^+^ macrophages in COCA and GBM were extremely low (Figure 2D,E). Macrophages were subsequently divided into AT and PT macrophages. NEAT1‐31^+^ macrophages were detected in all groups. The results indicated that AT‐macrophages were particularly enriched with NEAT1‐31 across the various cancers (Figure 2F). Patients receiving immunotherapy were enrolled, cancer samples were collected from patients receiving immunotherapy, and infiltrating lymphocytes were isolated. The ratio of NEAT1‐31^+^ macrophages was calculated using FACS; and the patients were divided into NEAT1‐31‐low and NEAT1‐31‐high groups, with the mean ratio of NEAT1‐31^+^ macrophages/CD45 cells serving as the cut‐off value. The response rate (RR) of ICB therapy for each cohort was calculated, and the RR was greater in the NEAT1‐31‐high cohort than that in the low NEAT1‐31‐low cohort. Thus, collectively, these findings revealed that *lincNEAT1* encodes NEAT1‐31, which is particularly enriched within antitumor macrophages and predicts a good ICB response across multiple cancers (Figure 2G).

### 2.3 NEAT1‐31 Promotes the Phagocytosis of Multiple Cancer Cells

**Figure 3:**
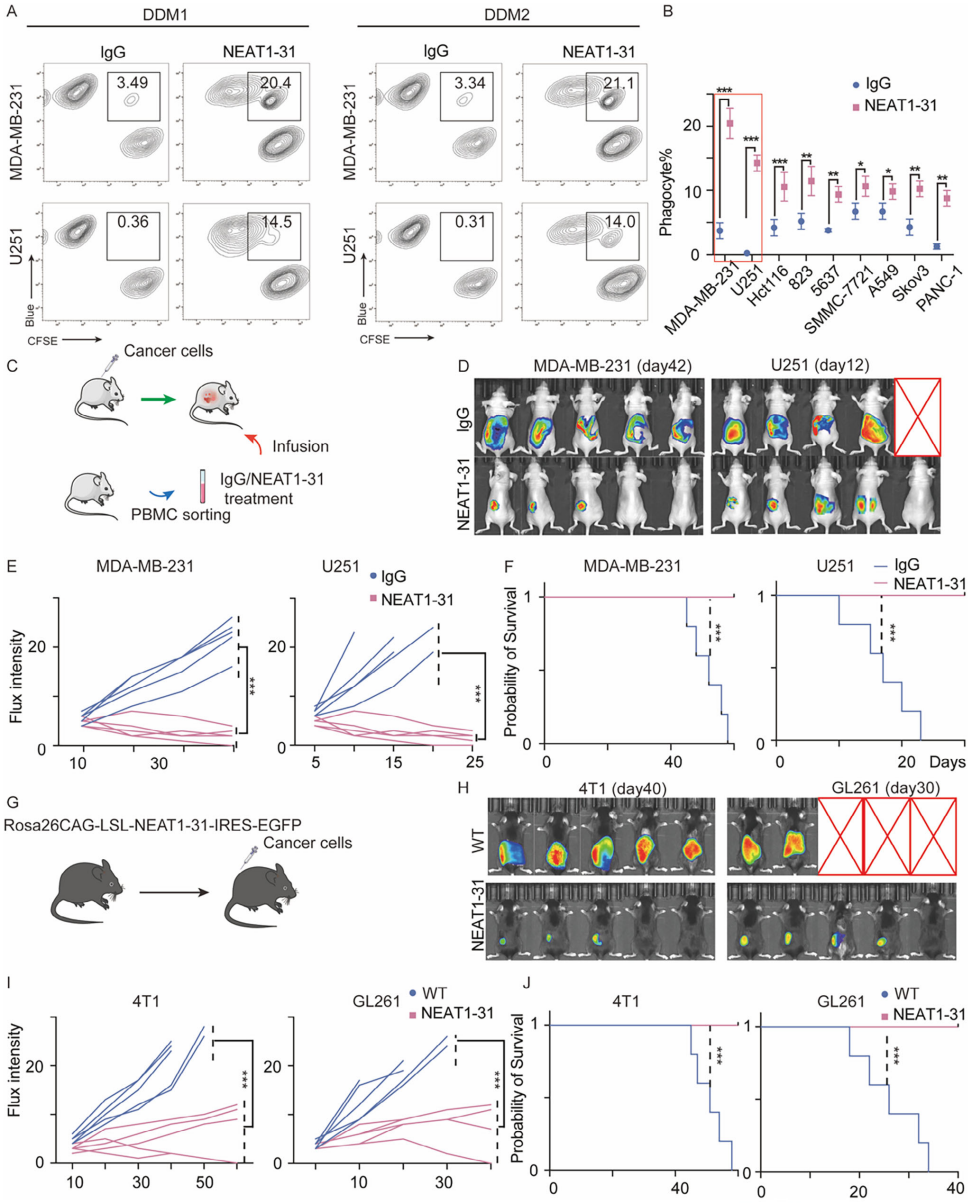
NEAT1‐31 promotes phagocytosis against multi cancer cell‐types. A) DDMs have been obtained from the peripheral blood of each donor and treated with IgG and the chemosynthetic NEAT1‐31 peptide, in vitro phagocytic assay has been performed. B) Phagocytosis of DDMs against multiple cancer cell‐types with IgG and chemosynthetic NEAT1‐31 peptide is depicted, Data are presented as means ± standard deviations (SDs); *n* = 3; unpaired *T*‐test; *** *p* < 0.001. C) Regarding the in vivo phagocytosis assay, macrophages have been collected and treated with IgG and chemosynthetic NEAT1‐31 peptide, macrophages have subsequently been infused back into tumor‐bearing mice. D) Mice bearing MDA‐MB‐231 and U‐251 cancer cells have been treated with IgG or NEAT1‐31, a representative image of the flux intensity has been obtained, *n* = 5 per group. E) Relative flux intensity of mice bearing MDA‐MB‐231 and U‐251 cancer cells with the indicated modifications are depicted, Animal = 5 per group, unpaired *T*‐test; *** *p* < 0.001. F) Overall survival time of mice with the indicated modifications are shown, Animal = 5 per group; log‐rank test; *** *p* < 0.001. G) In vivo phagocytosis assay involving NEAT1‐31 KI C57 mice is depicted. H) Representative image of the flux intensity, obtained from WT/NEAT‐31 KI mice, is shown, Animals = 5 per group. I) Relative flux intensity of mice‐bearing MDA‐MB‐231 and U‐251 cells with the indicated modifications is shown, Animals = 5 per group; unpaired *T*‐test; *** *p* < 0.001. J) Overall survival time of C57 mice with the indicated modifications is depicted, Animals = 5 per group; log‐rank test; *** *p* < 0.001. Abbreviations: CD = cluster of differentiation; PBMC = peripheral blood mononuclear cells; DDMs = donor‐derived macrophages; Ig = immunoglobulin; WT = wild‐type.

Two separate donor‐derived macrophages (DDMs) were collected and treated with chemosynthetic NEAT1‐31 peptide. NEAT1‐31 was removed, different macrophages were co‐incubated with the cancer cells, and phagocytosis was measured using a flow‐based phagocytosis assay. The results revealed that the application of the NEAT1‐31 peptide dramatically enhanced the phagocytosis of multiple cancer cell‐types. Furthermore, the phagocytosis of MDA‐MB‐231 and U‐251 GBM cell line cells was strongly enhanced (**Figure** **3**A,B; Figure S2A, Supporting Information). Therefore, we selected MDA‐MB‐231 and U‐251 cells for subsequent experiments. Antigen presentation is another critical function that promotes T‐cell recognition and cytotoxicity. Thus, we used T‐cell proliferation and chemokine detection assays to validate the antigen presentation ability of these cells. T‐cell function was strongly enhanced, as measured by an increased proliferation rate and production of interferon (IFN)‐γ (Figure S2B–E, Supporting Information). To assess the in vivo influence of the NEAT1‐31 peptide on phagocytosis, we used two xenograft models: a para‐orthotopic xenograft model, engrafted into the kidney capsule and additionally known as in vivo phagocytosis assay, and an in situ xenograft model. Cancer cells were transplanted into mice. Thereafter, mouse macrophages were sorted and induced from the blood, treated with immunoglobulin (Ig) G/NEAT1‐31 peptide, and infused back into the mice through the caudal vein (Figure 3C). NEAT1‐31 treatment resulted in tumor eradication, as measured by a decreased flux intensity and prolonged survival time (Figure 3D–F). In addition, this was confirmed with the in situ xenografts, as detected by impaired tumor progression and increased survival probability (Figure S2F–H, Supporting Information). Moreover, we established a NEAT1‐31 knock‐in (Rosa26CAG‐LSL‐Linc‐NEAT1‐IRES‐EGFP) model in C57 mice (Figure 3G). 100 µL containing 1 ×10^6^ MDA‐MB‐231‐Luc or 1 × 10^6^ U251‐Luc tumor cells were engrafted into the kidney capsule in situ, and the flux intensity and overall survival time were assessed (Figure 3H–J). Consistent with previous results, NEAT1‐31 promoted the elimination of multiple cancer cell types and prolonged overall survival (Figure S2I–K, Supporting Information).

### 2.4 NEAT1‐31 Promotes Aurora‐A Activity

**Figure 4:**
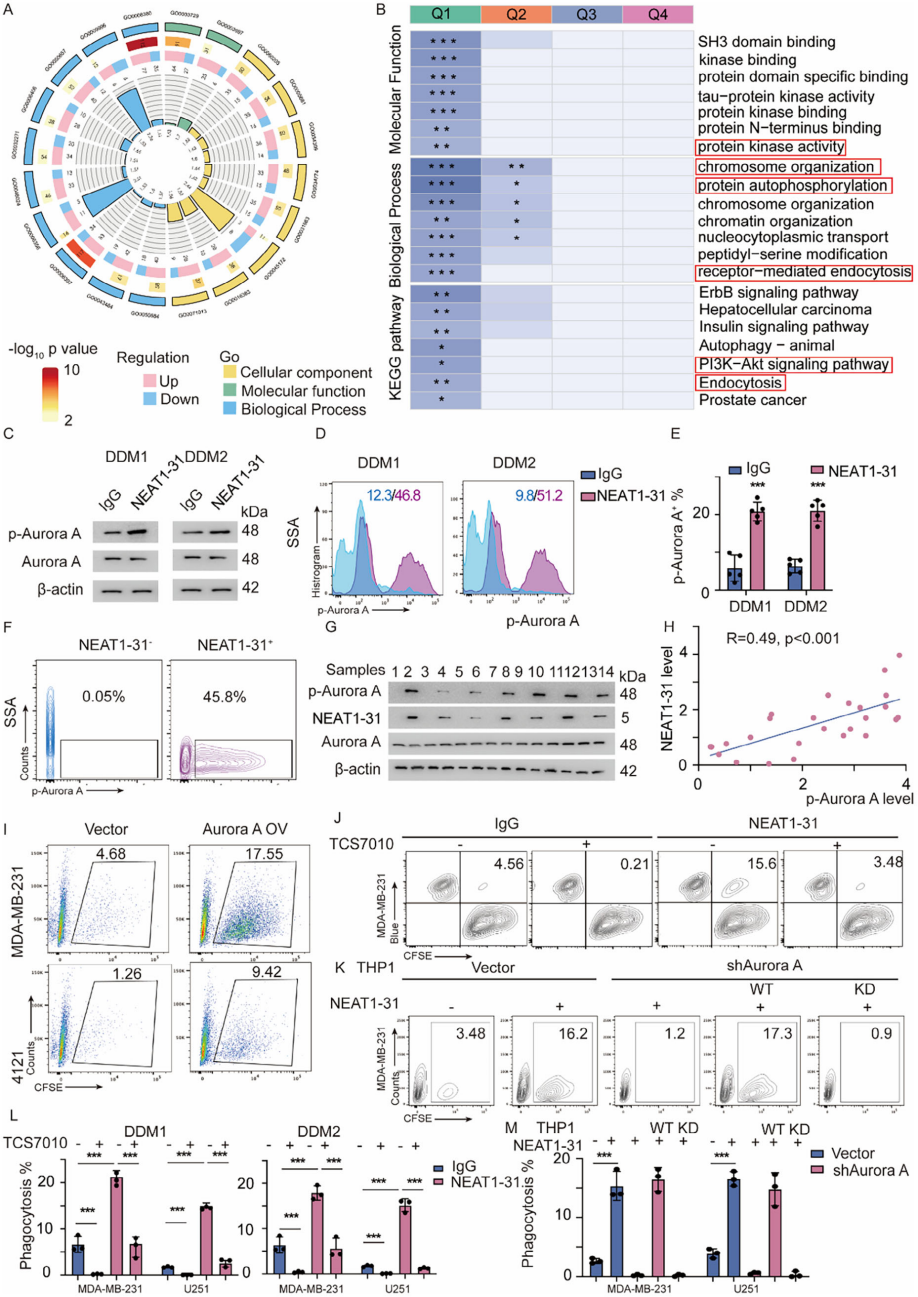
NEAT 1–31 promotes Aurora‐A activity. A) GO analysis of the DEPSs in NEAT1‐31 K.O. macrophages are depicted. NEAT1‐31 has been knocked out using the CRISPR/Cas9 system. Cells have been induced to differentiate into macrophages and have been subjected to phosphoproteomic analysis. B) DEPs have been divided into four groups (Q1–Q4), and the differentially enriched pathways, biological processes, and molecular functions have been analyzed. C) DDMs have been treated with IgG and NEAT1‐31, immunoblot was applied detecting the phosphorylation of Aurora A. D) Flow cytometry detecting the p‐Aurora‐A distribution in DDMs with indicated modifications is shown. E) Statistical analysis of the DDMs treated with IgG and NEAT1‐31 is shown, *n* = 5, data was presented as mean ± SD, unpaired *T*‐test, *** *p* <0.001. F) Macrophages derived from breast cancer specimens were divided into NEAT1‐31 −/+ subgroups, p‐Aurora A was detected using FACS, the representative image was present. G) Fourteen tumor‐infiltrated macrophages have been isolated and subjected to immunoblotting to detect p‐Aurora‐A and NEAT1‐31. H) The regression analysis of NEAT1‐31 and p‐Aurora A in G, regression score = 0.49, *p* < 0.001. I) THP1 cells were transfected with vector and Aurora A overexpression plasmid and were induced to macrophages, macrophages were subjected to in vitro phagocytosis assay. J) Aurora‐A‐specific inhibitor, TCS7010, has been applied to IgG and NEAT1‐31‐treated macrophages, and in vitro phagocytosis has been assessed. K) Aurora‐A has been knocked down in the THP1 cells, WT/KD alleles have been re‐expressed, and the cells have been collected and subjected to an in vitro phagocytosis assay. L) Statistical analysis of phagocytosis described in J is shown. *n* = 3; data are presented as means ± standard deviations (SDs), unpaired *T*‐test; *** *p* < 0.001. M) Statistical analysis of phagocytosis described in K is depicted, *n* = 3; data are presented as means ± standard deviations (SDs); unpaired *T*‐test; *** *p* < 0.001. Abbreviations: GO = gene ontology; CRISPR/Cas9 = clustered regularly interspaced short palindromic repeats/CRISPR‐associated protein 9; DEPs = differentially expressed proteins; DEPSs = differentially expressed phosphorylation sites; DDMs = donor‐derived macrophages; Ig = immunoglobulin; FACS = fluorescence‐activated cell sorting; THP1 = Tohoku Hospital Pediatrics‐1; WT = wild‐type; KD = kinase‐dead. All the experiments were repeated at least 3 times with similar results.

“Eat me” signaling is mediated by receptor‒ligand‐mediated‐ITAM activation and downstream activation of protein kinase C (PKC) and PI3K–AKT signaling.^[^ ^15^ ^]^ We have previously demonstrated that AT‐macrophages were particularly enriched with NEAT1‐31. Thus, we collected Tohoku Hospital Pediatrics‐1 (THP1) cells and treated them with PMA and LPS. *LincNEAT1* was knocked out using the clustered regularly interspaced short palindromic repeats (CRISPR)/CRISPR‐associated protein (Cas) 9 system. The cells were collected and subjected to phosphoproteomic analysis. Most peptide segments carried 2 to 3 charges; and the peptide length peaked at 7–20 amino acids, meeting the quality control requirements (Figure S3A, Supporting Information). Overall, 111 616 spectra were matched, 3514 proteins were identified, and 8700 peptides were modified (Figure S3B, Supporting Information). A total of 1236 differentially expressed proteins (DEPs) and 1868 differentially expressed phosphorylation sites (DEPSs) were identified, including 1047 upregulated DEPSs and 821 downregulated DEPSs (Figure S3C, Supporting Information). Gene ontology (GO) analysis was subsequently performed. Interestingly, DEPs were enriched in mitotic processes, spindle formation, phosphoprotein binding, and related mitotic cytokinetic arrangements (**Figure** **4**A). Macrophages are terminally differentiated cells without mitotic ability. We hypothesized that mitosis‐related proteins are involved in regulating ITAM activation. Therefore, we divided the DEPs into four groups (Q1–Q4) accordingly. A total of 361 modified sites were classified as Q1; whereas 460, 424, and 623 modified sites were classified as Q2–4, respectively (Figure S3D, Supporting Information). The results revealed that DEPs in Q1, characterized by the promotion of mitosis, were strongly correlated with protein kinase activity, endocytosis, and PI3K–AKT activation (Figure 4B). We hypothesize that it could be a protein that plays a significant role in the mitotic process that triggers the activation of the AKT pathway. This protein might have the feature of ectopic distribution and might possess phosphatase activity. Hence, we tested the activities of a series of proteins, such as Tau, AuroraA, etc. AuroraA exhibited a remarkable change in activity (Figure S4A, Supporting Information). For further confirmation, IB and FACS were used to assess the phosphorylation of Aurora‐A in DDM1/2 cells treated with the purified NEAT1‐31 peptide (Figure 4C–E). Macrophages from BRCA specimens were further isolated and probed for NEAT1‐31 and phosphorylated (p)‐Aurora‐A. The proportion of p‐Aurora‐A^+^ cells was elevated in NEAT1‐31^+^ macrophages (Figure 4F; Figure S4B, Supporting Information). IB was further performed to assess p‐Aurora‐A expression in DDMs, and regression analysis suggested that NEAT1‐31 was strongly correlated with p‐Aurora‐A (Figure 4G,H). Moreover, macrophages derived from paired normal tissues and tumors were collected and subjected to immunoblot to detect p‐Aurora A and p‐AKT. The results suggested that NEAT1‐31 was upregulated in normal tissue derived macrophages and the expression of NEAT1‐31 is conspicuously correlated with the phosphorylation levels of Aurora A and AKT (Figure S4C, Supporting Information). THP1 cells were transfected with an Aurora A plasmid and induced to macrophages. The in vitro phagocytosis assay suggested increased “eating” of multiple cancer cell‐types (Figure 4I; Figure S4D, Supporting Information). To validate the biological importance of NEAT1‐31‐mediated activation of Aurora A‐in phagocytosis, the Aurora‐A‐specific inhibitor, TCS7010, was applied to NEAT1‐31 treated DDMs. IB was performed to assess p‐Aurora‐A expression (Figure S4E, Supporting Information). FACS was performed to evaluate the phagocytic ability. The results demonstrated that phagocytosis was inhibited in DDMs treated with TCS7010. Moreover, regarding NEAT1‐31 treated DDMs, TCS7010 reversed the increase in phagocytosis to baseline levels (Figure 4J,L; Figure S4F,G, Supporting Information). Aurora‐A activity was assessed based on the phosphorylation of specific tyrosine residues. We re‐expressed wild‐type (WT) Aurora‐A and the kinase‐dead (KD) allele thereof, in THP1/U‐937‐induced macrophages using a lentivirus. The phosphorylation of Aurora‐A and the phagocytosis rate were analyzed (Figure S4H, Supporting Information). As expected, NEAT1‐31 promoted the phosphorylation of Aurora‐A and phagocytosis of WT macrophages, however did not promote the phosphorylation of Aurora‐A or phagocytosis of KD macrophages (Figure 4K,M; Figure S4I, Supporting Information). Collectively, these findings showed that NEAT1‐31‐mediated activation of Aurora‐A is critical for promoting phagocytosis.

### 2.5 NEAT1‐31 Directly Binds to Aurora‐A

**Figure 5:**
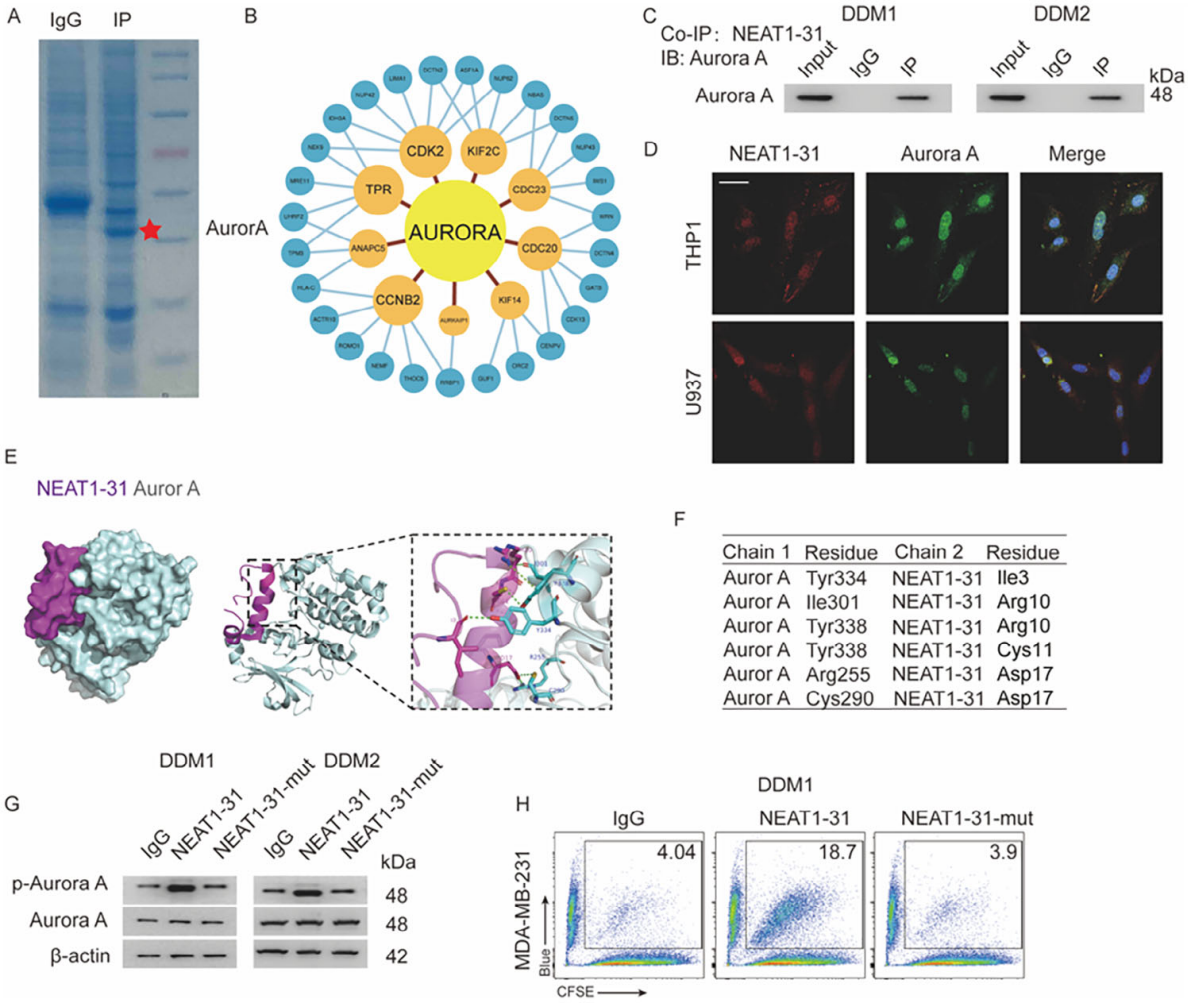
NEAT1‐31 binds directly with Aurora‐A. A) Coomassie blue staining of massive co‐IP using the NEAT1‐31 antibody in macrophages is depicted. B) Protein interacting network analysis of the potential interacting proteins is shown. C) IP–IB assay for detecting NEAT1‐31 and Aurora‐A, using antibodies. D) Immunofluorescence of NEAT1‐31 and Aurora A in THP1 and U937 cells. E) Molecular docking has been applied, and the interacting residues are demonstrated. F) Detailed image of the interacting residues of Aurora‐A and NEAT 1–31 is shown. G) Purified mutant protein has been generated: NEAT1‐31N17R, referred to as NEAT1‐31‐mut. DDM1/2 cells have been treated with the WT or NEAT1‐31‐mut. The expression of p‐Aurora‐A has been assessed by immunoblotting. H) DDM1 cells have been treated with IgG, NEAT1‐31, or NEAT1‐31‐mut, in vitro phagocytic assay has been performed. Abbreviations: cys = cysteine; tyr = tyrosine; ile = isoleucine; arg = arginine; asp = aspartic acid; kDa =; NEAT1 = nuclear enriched abundant transcript 1; co‐IP = co‐immunoprecipitation; IP–IB assay = Immunoprecipitation to immunoblotting assay; DDMs = donor‐derived macrophages; WT = wild‐type; Ig = immunoglobulin. All the experiments were repeated at least 3 times with similar results.

Multiple kinases regulate Aurora‐A activity.^[^ ^16^ ^]^ To uncover the potential underlying mechanism involved, a co‐immunoprecipitation (co‐IP) assay was performed. Additionally, a liquid chromatography–mass spectrometry (LC–MS) analysis was used to identify the potential interacting proteins (**Figure** **5**A: Aurora‐A is labeled). A protein interaction network was constructed to identify the core interacting proteins. Aurora‐A was the hub of the network, and nine binding partners were identified in the same dataset (Figure 5B). This was further confirmed by the immunoprecipitation to IB (IP–IB) assay and immunofluence assay (Figure 5C,D; Figure S4J, Supporting Information). Molecular docking was performed to predict potential interacting residues in NEAT1‐31 and Aurora‐A. A Ramachandran plot was generated, which indicated that the 3D structure of the model was reasonable. The interacting residues are shown in detail (Figure 5E,F). A mutant allele was established: NEAT1‐31N17R, referred to as NEAT1‐31‐mut. DDM1/2 cells were treated with purified WT or the NEAT1‐31‐mut. The expression of p‐Aurora‐A was assessed by IB. WT NEAT1‐31 promoted Aurora‐A activation, while the NEAT1‐31‐mut failed to activate Aurora‐A (Figure 5F). An in vitro phagocytosis assay was performed to confirm the effects of NEAT1‐31‐mut on phagocytosis. The percentage of phagocytic macrophages was completely reversed to baseline levels in cells treated with NEAT1‐31‐mut (Figure 5G,H; Figure S4K,L, Supporting Information).

### 2.6 NEAT1‐31‐Mediated Phosphorylation of Aurora‐A Activates the PI3K–AKT Pathway

**Figure 6:**
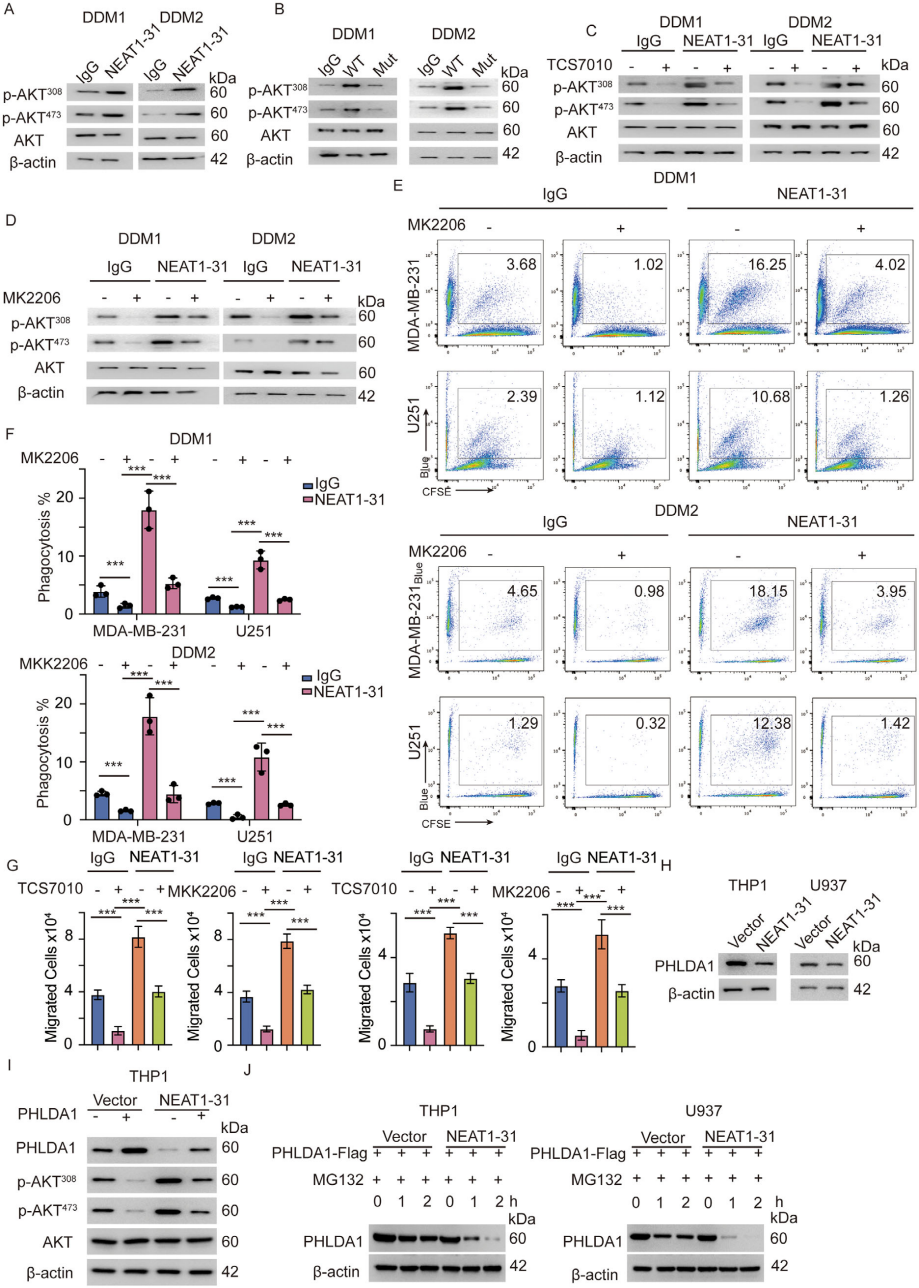
NEAT1‐31 mediated Aurora‐A activation promotes the activation of the PI3K–AKT pathway. A) DDMs were treated with NEAT1‐31 and subjected to immunoblot detecting p‐AKT, DDM, donor derived macrophage. B) Immunoblot of p‐AKT in cells with indicated modifications, DDM, donor derived macrophage. C) DDMs were treated with NEAT1‐31 and Aurora A specific inhibitor TCS7010, p‐AKT was detected using immunoblot, DDM, donor derived macrophage. D) AKT inhibitor MK2206 was applied in different cells, p‐AKT was detected, DDM, donor derived macrophage. E) In vitro phagocytosis analysis of DDMs with indicated modifications, DDM, donor derived macrophage. F) The statistical analysis of E, *n* = 3, data was presented as mean ± SD, Kruskal–Wallis test, Dunn's post hoc test, *** *p* < 0.001, DDM, donor derived macrophage. G) The migration assay was applied in cells with indicated modifications, migrated cells were measured and analyzed, *n* = 3, data was presented as mean ± SD, Kruskal–Wallis test, Dunn's post hoc test, *** *p* < 0.001. H) Immunoblot detecting PHLDA1 in cells with indicated modifications. I) PHLDA1 was re‐expressed in NEAT1‐31 overexpression cells, p‐AKT was detected using immunoblot. J) THP1 and U937 were transfected with PHLDA1 and treated with MG132, cells at different time point were collected and subjected to immunoblot detecting PHLDA1. Abbreviations: NEAT1 = nuclear enriched abundant transcript1; PI3K–AKT = phosphatidylinositol 3‐kinase/protein kinase B; DDMs = donor‐derived macrophages; IB = immunoblotting; PHLDA1 = pleckstrin homology‐like domain family A, member 1. All the experiments have been repeated at least thrice with similar results.

Activation of Aurora‐A has not been shown to directly enhance phagocytosis in macrophages. Aurora‐A promotes the phosphorylation of downstream genes, such as those in the mitogen‐activated protein kinase (MAPK) and PI3K–AKT pathways in cancers, and plays vital roles in multiple biological processes.^[^ ^17^, ^18^ ^]^ PI3K–AKT pathway activation is a hallmark of the activation of ITAMs. Combined with previous results, the activation of Aurora‐A was correlated with the PI3K–AKT pathway (Figure 4B). Therefore, we assessed PI3K–AKT signaling in DDMs treated with NEAT1‐31 or the NEAT1‐31‐mut. Protein kinase B (AKT) phosphorylation at threonine 308 (T308) and serine 473 (S473) increased in the NEAT1‐31‐treated cells, however not in the NEAT1‐31‐mut‐treated cells (**Figure** **6**A,B). To validate the biological importance of Aurora‐A in regulating PI3K–AKT signaling and phagocytosis, we treated cells with TCS7010 and assessed AKT phosphorylation. TCS7010 inhibited Aurora‐A phosphorylation and reversed Aurora‐A‐mediated AKT activation (Figure 6C). The specific AKT inhibitor, MK2206, was applied.^[^ ^19^ ^]^ Moreover, an in vivo phagocytosis assay and FACS analysis were performed to determine the importance of PI3K–AKT signaling in phagocytosis. MK2206 inhibited PI3K–AKT pathway activation and phagocytosis; completely reversing NEAT1‐31‐mediated AKT activation and phagocytosis promotion (Figure 6D–F). PI3K–AKT activation promotes cytoskeletal rearrangement and enhances macrophage migration of macrophages.^[^ ^20^ ^]^ A migration assay was performed on DDMs with the indicated modifications. NEAT1‐31 promoted DDM migration, while TCS7010 and MK2206 inhibited NEAT1‐31‐mediated migration (Figure 6G). The mechanism by which Aurora‐A activates AKT has not been fully elucidated; however, studies have revealed that Aurora‐A activation promotes the degradation of pleckstrin homology‐like domain family A, member 1 (PHLDA1), an AKT suppressor.^[^ ^21^ ^]^ To confirm this, first, we detected PHLDA1 in NEAT1‐31‐overexpressing THP1/U‐937 cells. As predicted, PHLDA1 was downregulated and re‐expression of PHLDA1 inhibited AKT activation (Figure 6H,I). After different macrophages were transfected with FLAG‐tagged PHLDA1 and treated with MG132, the cells were collected at different time‐points and subjected to IB. The degradation of PHLDA1 was dramatically enhanced in NEAT1‐31‐treated DDMs (Figure 6J).

### 2.7 NEAT1‐31 Enhanced the Efficacy of Anti‐CD47 Therapy In Vitro and In Vivo

**Figure 7:**
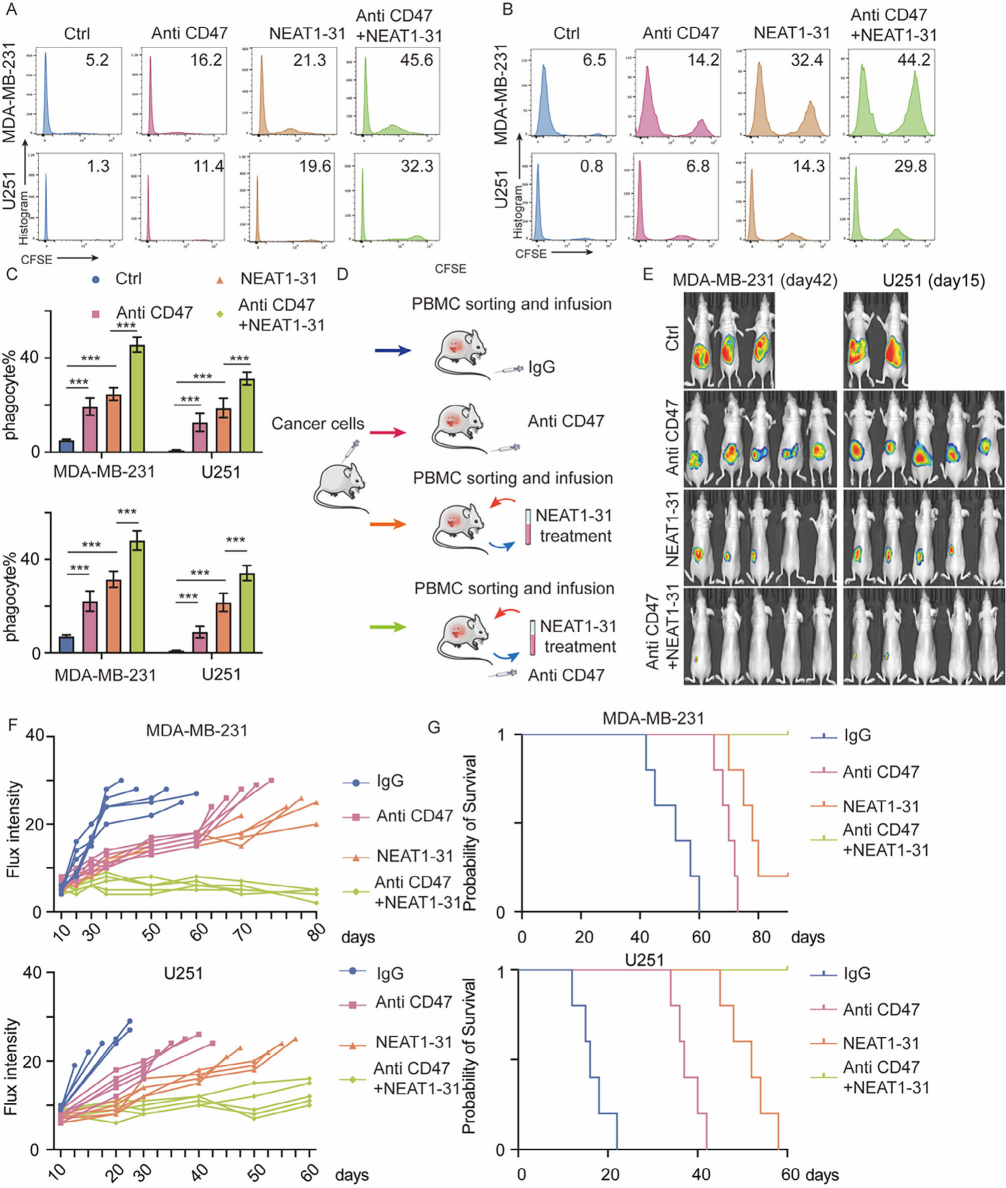
NEAT1‐31 promotes anti‐CD47 efficacy. A,B) DDMs were treated with NEAT1‐31, anti CD47 antibody or in combination. In vitro phagocytosis was applied, phagocyte was detected using FACS. C) The statistical analysis of A,B, *n* = 3, data was presented as mean ± SD, Kruskal–Wallis test, Dunn's post hoc test, *** *p* < 0.001. D) The illustration of the combined therapy strategy, mice were treated with anti CD47 antibody or NEAT1‐31 treated macrophage. E) The flux intensity of mice bearing MDA‐MB‐231 and U251 with indicated modifications, *n* = 5 per group. F) The statistical analysis of Flux intensity in mice with indicated modifications, *n* = 5 per group. G) The overall survival time of mice with indicated modifications, *n* = 5 per group. Abbreviations: NEAT = nuclear enriched abundant transcript; DDMs = donor‐derived macrophages; FACS = fluorescence‐activated cell sorting; CD = cluster of differentiation. All the experiments were repeated at least 3 times with similar results.

**Figure 8:**
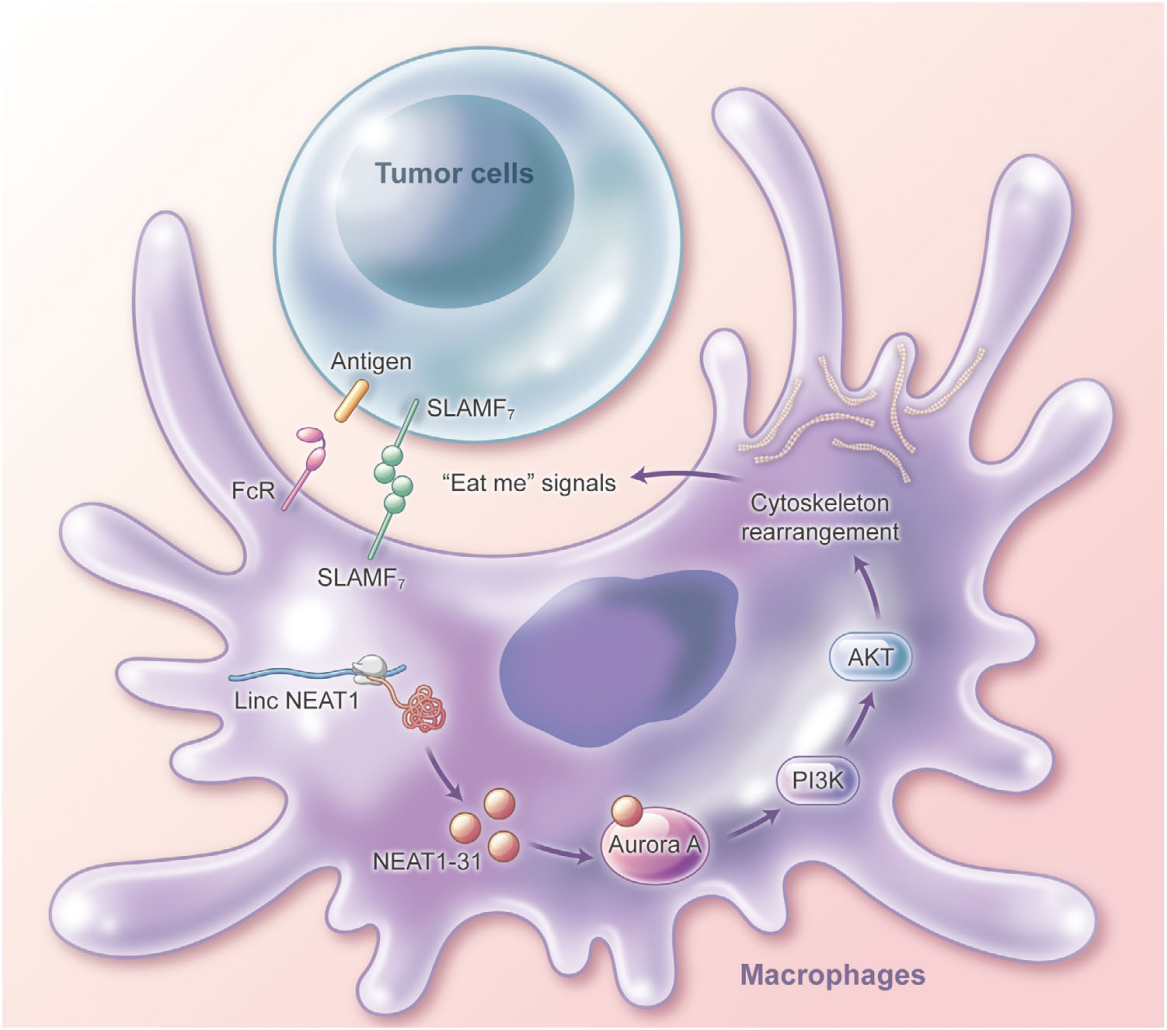
Graphical abstract of NEAT1‐31 on phagocytosis.

CD47 is one of the most important phagocytic immune checkpoints, and the specific blockade of CD47 via an antibody promotes phagocytosis. To verify the clinical application of NEAT1‐31 in promoting phagocytosis, we treated DDMs with the NEAT1‐31 peptide with or without a CD47 antibody. The cells were subjected to a phagocytosis assay, and the results revealed that NEAT1‐31 and an anti‐CD47 antibody promoted phagocytosis, as was hypothesized; and that NEAT1‐31 and treatment with an anti‐CD47 antibody had synergistic effects (**Figure** **7**A–C). Subsequently, a para‐orthotopic xenograft model was established. Mice were treated with IgG or anti‐CD47 antibodies, and PBMCs were sorted and treated with NEAT1‐31. The flux intensity was assessed and the overall survival time was calculated. Moreover, NEAT1‐31 and anti‐CD47 promoted the elimination of cancer cells and prolonged the survival time of mice with BRCA and GBM (Figure 7D–G). These findings were further confirmed using a C57 model, in which NEAT1‐31 and anti‐CD47 antibodies inhibited tumor progression through a synergistic effect (Figure S5A–F, Supporting Information). Overall, our results showed that *lncNEAT1* translated micropeptide NEAT1‐31 promotes macrophage phagocytosis, by direct binding with Aurora‐A (**Figure** **8**).

## 3 Discussion

Given that macrophages and phagocytic macrophages play key roles in adaptive immunity, the identification of phagocytosis‐associated checkpoints is one of the most popular research fields worldwide. With these advances, studies have identified potential target sites, such as CD47, Siglec7, and CD24. Furthermore, the range of potential target sites based on tumor specificity has been narrowed.^[^ ^6^, ^8^ ^]^ Currently, no systematic scanning strategy is available, possibly due to the lack of phagocytic macrophage induction methods. Theoretically, phagocytosis actually involves two cell types, with phagocytic macrophages “eating” cancer cells. However, distinguishing phagocytic macrophages from cancer cells is challenging. Studies have attempted to identify potential checkpoints in mice by using CRISPR‐Cas9.^[^ ^22^ ^]^ This approach has helped identify the “don't eat me” signaling molecules expressed on the surface of cancer cells. The mechanisms of phagocytosis in mice and humans are not entirely identical, and the phagocytosis of heterotypic and homotypic tumor cells is completely different. In the present study, we collected macrophages from healthy donors and performed an in vitro phagocytosis assay. The phagocytic macrophages were sorted and subjected to ribosomal profiling. First, we identified phagocytic macrophage‐specific genes by comparing phagocytic macrophages with cancer cells (FPKM = 0 for MDA‐MB‐231 cells; FPKM > 1 for phagocytic macrophages). Second, we compared the expression of phagocytic macrophage‐specific genes in phagocytic macrophages and macrophages, which ultimately led to the identification of phagocytic activators. Undoubtedly, this approach will lead to the omission of a portion of phagocytic macrophage activators that are commonly expressed in cancer cells and phagocytic macrophages; however, this approach still provides reliable targets. Thus, we selected *lincNEAT1* for further investigation. Moreover, in vivo and in vitro phagocytosis assays confirmed the function of *lincNEAT1*, which encodes for NEAT1‐31, in promoting phagocytosis. Thus, our ribosome profiling and scanning strategies were shown to be highly reliable.

Long non‐coding RNAs are defined as autonomously transcribed RNAs longer than 200 nucleotides with minimal coding potential.^[^ ^23^ ^]^ More than half do not overlap with annotated coding genes and are called long intergenic noncoding RNAs *(lincRNAs)*.^[^ ^24^ ^]^ Due to the lack of overlap between *lincRNAs* and the coding transcripts of their annotated genes, *lincRNAs* exert their biofunctions mainly by regulating the expression of hub genes through multiple pathways. These biofunctions include decoying microRNAs, modulating the binding of transcription factors and promoters, working as scaffolds between interacting proteins, and epigenetically modulating chromatin.^[^ ^25^ ^]^ However, once termed “non‐coding RNAs”, recent evidence has suggested that a small fraction of *lincRNAs* have small open reading frames (*smORFs*) and may translate into functional proteins.^[^ ^26^, ^27^ ^]^ The microprotein, small regulatory polypeptide of amino acid response, is encoded by *LINC0091* and inhibits the activation of mammalian target of rapamycin complex 1 and muscle regeneration.^[^ ^28^ ^]^ Myoregulin is a *LINC00948*‐encoded micropeptide exclusively expressed in skeletal muscles that inhibits calcium uptake in the sarcoplasmic reticulum through direct interaction with sarcoendoplasmic reticulum calcium ATPase.^[^ ^29^ ^]^ Many additional *smORF*‐encoded micropeptides have been identified through ribosome profiling with deep sequencing of mRNA fragments, or Ribo‐seq, and proteomic approaches.^[^ ^30^, ^31^, ^32^ ^]^ Because the sequences of *smORFs* are more conserved than those of introns, and *smORF*‐harboring *lincRNAs* are expressed at higher levels than that of *lincRNAs* without *smORFs*, the translated microproteins may perform broader functions than non‐coding *lincRNAs*.^[^ ^27^, ^33^ ^]^

“Eat me” and “don't eat me” signals are generated by activating ITAMs or immunoreceptor tyrosine‐inhibiting motifs (ITIMs), respectively.^[^ ^34^, ^35^ ^]^ This process is ligand–receptor‐specific, in which receptors bind to active ITAMs or ITIMs, ultimately promoting cytoskeletal rearrangement. In our study, we used proteomics and phosphoproteomics to reveal that NEAT1‐31 promotes Aurora‐A activity and the downstream PI3K–AKT pathway. Ligand‒receptor‐mediated phagocytosis is nonselective. By contrast, phagocytosis is the result of the integration of “eat me” and “don't eat me” signals. Concerning normal cells, “don't eat me” signals are predominant, and “eat me” signals are barely activated. Regarding cancer cells, “don't eat me” signals are intensively activated, while “eat me” signals are additionally activated. We hypothesized that NEAT1‐31‐mediated activation of PI3K–AKT enhances phagocytosis in macrophages that recognize cancer cells, however not in macrophages that are not activated by tumor ligands. However, the adverse events and risks associated with autoimmune diseases require further investigation.

Aurora‐A plays a key role in chromosomal arrangement and spindle formation.^[^ ^36^ ^]^ Moreover, Aurora‐A performs a vital function in pathological and physiological progression.^[^ ^37^ ^]^ Aurora‐A expression is appreciably upregulated in cancer and promotes tumorigenesis and cancer progression through various means.^[^ ^16^, ^38^, ^39^ ^]^ One of the most important pathways is the activation of PI3K–AKT signaling. However, the underlying mechanisms remain unclear. Additionally, the biological importance of Aurora‐A in phagocytosis has not been elucidated. We were the first to detect the phosphorylation of Aurora‐A in various macrophages and revealed that Aurora‐A activation promotes phagocytosis. Furthermore, we overexpressed Aurora‐A in macrophages. Aurora‐A overexpression promotes phagocytosis of multiple cancer cell‐types. Furthermore, we overexpressed Aurora‐A in cells using a PI3K–AKT‐specific inhibitor. NEAT1‐31‐mediated phagocytosis was reversed.

Our study indicates that NEAT1‐31 promotes the endocytosis of macrophages via the Aurora A and PI3K‐AKT pathways. In tumor cells, this pathway also plays a crucial role in promoting tumor progression, which makes it impossible to directly administer the polypeptide for treatment. Instead, as stated in the article, macrophages need to be isolated and treated with NEAT1‐31 before being re‐infused. This process may potentially cause excessive activation of the inherent immune function of macrophages, thereby triggering a series of issues, such as autoimmune diseases. Simultaneously, as NEAT1‐31 does not function in a receptor‐ligand manner. This restricts NEAT1‐31 to promoting the phagocytic function of only those macrophages that have recognized cancer cells. If tumor cells highly express the “don't eat me” signaling, NEAT1‐31 might have an ineffective outcome. This makes it advisable for NEAT1‐31 to be used in combination with other treatments, such as anti‐PD1/PDL1, etc.

In conclusion, we established an in vitro phagocytosis assay using donor macrophages and subjected the phagocytic macrophages to ribosome profiling. *LincNEAT1* is specifically expressed in phagocytic macrophages. The micropeptide encoded by *LincNEAT1* is termed, “NEAT1‐31”. Using FLAG‐tagged NEAT1‐31 expressed in HEK293 T‐cells, a specific antibody was developed for NEAT1‐31. NEAT1‐31 promoted phagocytosis in vivo and in vitro by activating the Aurora‐A–PI3K–AKT pathway, and NEAT1‐31 enhanced the efficacy of anti‐CD47 therapy.

## 4 Experimental Section

### Healthy Donors Recruitments, Tumor Specimen, and Ethical Approval

Healthy donors were recruited from the Guangzhou Women and Children's Medical Center. Peripheral blood samples (20 mL per donor) were collected for subsequent in vitro phagocytosis assays. Prior to sample collection, all donors provided written informed consent after receiving a detailed explanation of the study's purpose and procedures. Ethical approval for this study was granted by the Institutional Review Board (IRB) of the Guangzhou Women and Children's Medical Center (Reference Number: 060A01). Additionally, written informed consent was obtained from all participating healthy donors and patients. This study was conducted in full compliance with ethical guidelines governing research involving human participants, including those outlined in the Declaration of Helsinki.

### Monocyte Isolation

Peripheral blood from donors was collected and monocytes were isolated using EasySep Direct Human Monocyte Isolation Kit following the manufacture's protocol. Briefly, peripheral blood cells except monocytes were washed through binding with the beads. Monocytes were harvested and subjected to in vitro inductions.

### In Vitro Phagocytosis Assay

Peripheral blood samples were collected from each donor, and monocytes were isolated using the EasySep Direct Human Monocyte Isolation Kit (STEMCELL Technologies, Catalog #19669RF) according to the manufacturer's protocol. Monocytes were then differentiated into macrophages by stimulation with phorbol 12‐myristate 13‐acetate (PMA, 100 ng mL^−1^). For cell labeling, macrophages were stained with CellTracker CMF2HC (Catalog #C12881), while MDA‐MB‐231 breast cancer cells were labeled with CellTrace CFSE (Catalog #C34570). After trypsinization, cells were washed once with PBS and resuspended in pre‐warmed PBS (37 °C). The staining solution was prepared at a working concentration of 5 µM, and cells were incubated at 37 °C for 30 min. Following incubation, cells were washed once with PBS and once with culture medium before further processing. Following labeling, macrophages and MDA‐MB‐231 cells were co‐cultured for 2 h, after which the cells were harvested and analyzed by fluorescence‐activated cell sorting (FACS). Cells that exhibited dual fluorescence signals for CFSE and CMF2HC were identified as phagocytic macrophages, those labeled only with CMF2HC were classified as macrophages, and cells labeled solely with CFSE were confirmed as MDA‐MB‐231 cells. Equal numbers of phagocytic macrophages, macrophages, and MDA‐MB‐231 cells were collected and subsequently subjected to ribosome profiling for further analysis.

### Generation of NEAT1‐31 Antibody

The NEAT1‐31‐specific antibody was custom‐generated by Hua'an Biotechnology (Hangzhou, China). To produce the antibody, the NEAT1‐31 peptide was initially conjugated to keyhole limpet hemocyanin (KLH) and bovine serum albumin (BSA). The conjugated peptide was then used as an immunogen and administered via multiple subcutaneous injections to New Zealand White rabbits to elicit an immune response. Following immunization, blood samples were collected from the rabbits, and the presence of NEAT1‐31‐specific antibodies in the serum was assessed using enzyme‐linked immunosorbent assay (ELISA) and Western blot analysis. Finally, the antibodies were purified using a non‐phosphopeptide affinity column to ensure specificity and purity before further application.

### Flow Cytometry

Samples were prepared and resuspended in staining buffer, after incubating with fluorescence labelled primary antibody. Samples were subjected to BD FACSymphony A3 for flow cytometry analysis. Cells are initially gated on forward scatter (FSC) and side scatter (SSC) to define the overall population. Doublets are excluded by comparing FSC‐A and FSC‐H. Positive cell populations are then determined using appropriate isotype controls and unstained samples to set the threshold. Both unstained samples and isotype control antibodies were used to ensure accurate gating and to validate the specificity of the antibody staining. Following primary antibody was applied: NEAT1‐31 (this study), CD80 (abcam, ab283655) and CD206 (BD Pharmingen, 551135).

### Ribosome Profiling

The ribosome profiling technique was carried out as previously described^42^. After obtaining ribosome‐protected fragments (RPFs), libraries for ribosomal profiling were constructed using the NEBNext Multiple Small RNA Library Prep Set for Illumina (E7300S, E7300L) according to the manufacturer's protocol. Briefly, adapters were ligated to both ends of the ribosome footprints to facilitate subsequent processing. Reverse transcription was then performed to convert the RNA fragments into complementary DNA (cDNA), followed by PCR amplification to enrich for the target sequences. PCR products in the 140–160 bp size range, which represent the expected size of the ribosomal profiling library, were selected and purified. The final cDNA library was then sequenced using the Illumina HiSeq X10 platform. This approach ensured the generation of high‐quality sequencing data for detailed analysis of ribosome occupancy and translation dynamics.

### Metagene Analysis and Identification of Differential Expressed Genes

Low‐quality reads were filtered using FASTP (version 19). The short‐read alignment tool Bowtie2 was employed to map the reads to a ribosomal RNA and transfer RNA database. Reads that mapped to ribosomal RNA and transfer RNA were then removed. The remaining high‐quality reads from each sample were subsequently aligned to the reference genome using Bowtie2, with no mismatches allowed during the mapping process. To visualize the ribosome footprints (RFs) around the start and stop codons of metagenes, read counts were determined at each position along every gene. These read counts were then aggregated across all genes to generate a comprehensive overview. Metagene plots were created using R (https://www.r‐project.org/), with the read counts and coding sequence (CDS) boundaries from the transcript coordinates as input data. For identifying differentially expressed genes (DEGs) between phagocytes and macrophages, we first defined phagocyte‐specific genes by comparing phagocytes with cancer cells (FPKM = 0 in MDA‐MB‐231 cells, FPKM >1 in phagocytes). We then compared these phagocyte‐specific genes to those expressed in macrophages. Finally, DEGs between phagocytes and macrophages were identified by applying a fold change threshold of 2 and selecting genes with *p* < 0.01.

### In Vivo Phagocytosis Assay and Animal Experiments

For the para‐orthotopic experiments, 4‐5‐week‐old female athymic nude mice were purchased. Five mice were randomly assigned to each experimental group. 100 µL of a suspension containing 1 × 10⁶ MDA‐MB‐231‐Luc or 1 × 10⁶ U251‐Luc tumor cells was implanted into the renal capsule of each mouse. Tumor‐bearing mice were treated with either monocyte infusion or anti‐CD47 antibody. Monocytes were isolated from the mice and treated with purified NEAT1‐31 protein before being infused through the caudal vein. In parallel, 400 µg of anti‐CD47 antibody (clone B6.H12, Bio X Cell) was injected intraperitoneally into the mice every other day. The growth of tumor xenografts was monitored using bioluminescence imaging (BLI) with an IVIS machine (PhotoSound PAFT/256), and the relative flux intensity was measured to assess tumor progression. The overall survival time of the mice was also recorded and analyzed. All animal studies were approved by the Ruiye Model Animal (Guangzhou) Biotechnology Co., Ltd Experimental Animal Ethics Committee (approval number: RYEth‐20230615261). These studies adhered to the WMA Statement on Animal Use in Biomedical Research and the EU Directive 2010/63/EU on the protection of animals used for scientific purposes.

### Generation of the NEAT1‐31 KI Mouse Model

The Rosa26CAG–LSL‐Linc‐neat1‐IRES‐EGFP mouse model was developed by Shanghai Model Organisms Center, Inc. This model was generated using the CRISPR/Cas9 system in a C57BL/6J mouse background. Briefly, a targeting vector containing the following components was constructed: CAG‐loxP‐STOP‐loxP‐Kozak‐Linc‐neat1‐IRES‐EGFP‐woodchuck hepatitis virus post‐transcriptional regulatory element (WPRE)‐bGH poly(A). Cas9 mRNA was in vitro transcribed using the mMESSAGE mMACHINE T7 Ultra Kit (Ambion, TX, USA) according to the manufacturer's instructions and then purified with the MEGAclear Kit (Thermo Fisher, USA). The F0 mice were then crossed with C57BL/6J mice to generate Rosa26CAG–LSL‐Linc‐neat1‐IRES‐EGFP heterozygous mice. For tumor establishment, T1‐Luc and GL261‐Luc cancer cells were injected either into the renal capsule or directly into the tumor site. Tumor growth was monitored using bioluminescence imaging (BLI) with an IVIS imaging system (PhotoSound PAFT/256), and relative flux intensity was measured. The overall survival time of the mice was recorded and analyzed for further evaluation. This model serves as an effective tool for studying Linc‐neat1 gene expression and its impact on tumor progression in vivo.

### Molecular Docking

The 3D structure of the Aurora A protein was downloaded from RCSB Protein Data Bank (PDB ID: 4UV7). Protein–protein docking in ClusPro server4‐8 (https://cluspro.org) was used for molecular‐docking simulations of NEAT1‐31 and for predicting the binding affinity to Aurora A. First, preprocess the protein and small molecule by removing water and adding hydrogen atoms, as these factors influence the calculation of interactions during docking. Next, define the docking pocket and set the docking parameters. AutoDock employs a semi‐flexible docking approach, where the protein remains rigid while the small molecule retains a degree of structural flexibility, exploring the protein surface to identify potential binding regions. Save the configured docking files and perform docking. Among multiple docking results, select the one with the highest binding affinity (i.e., the lowest binding energy) as the final outcome. The reliability of the docking results can be evaluated based on binding energy (more negative values indicate stronger binding), the number of hydrogen bonds (which contribute to binding strength), and the root mean square deviation (RMSD) of the docked poses (lower values indicate higher accuracy, typically <2 Å is required). Finally, export the docked complex and visualize it using PyMOL for further structural analysis.

### Western Blot

Cultured cells or patient tissue samples are lysed in lysis buffer supplemented with protease and phosphatase inhibitors, followed by ultrasonication or vortex mixing. The lysates are centrifuged at 12000 × g for 10 min at 4 °C, and the supernatant is collected for protein quantification using the BCA assay. Equal amounts of protein (30–50 µg) are mixed with 5× SDS loading buffer and denatured at 95 °C for 5 min. The samples are then loaded onto a 10–12% polyacrylamide gel and subjected to SDS‐PAGE under a constant voltage of 80 V for stacking and 120 V for resolving until the bromophenol blue dye front reaches the bottom of the gel. Proteins are transferred onto a PVDF membrane using the wet transfer method, with transfer parameters adjusted based on molecular weight. Membranes are blocked with 5% nonfat milk prepared in TBST at room temperature for 1 h, followed by incubation with the primary antibody (at an appropriate dilution) overnight at 4 °C. After washing, membranes are incubated with an HRP‐conjugated secondary antibody at room temperature for 1 h. Following extensive washing, protein bands are visualized using an enhanced chemiluminescence (ECL) detection system and imaged accordingly.

### Immunoprecipitation

Cells were lysed in ice‐cold lysis buffer (0.3% CHAPS, 10 mM β‐glycerol phosphate, 10 mM pyrophosphate, 40 mM HEPES (pH 7.4), 2.5 mM MgCl2 and EDTA‐free protease inhibitor). After complete lysis, the lysates are centrifuged at 12000 × g for 10 min at 4 °C, and the supernatant is collected for further analysis. An appropriate amount of protein (500–1000 µg) is incubated with a suitable concentration of Neat‐31 antibody or Aurora A. Pre‐equilibrated Protein A/G magnetic beads or agarose beads are then added, followed by incubation at 4 °C under gentle rotation for 8–12 h to allow the formation of immune complexes. The immunoprecipitates are washed three times with PBST to remove nonspecifically bound proteins. The purified complexes are subsequently subjected to Western blot analysis and liquid chromatography‐mass spectrometry (LC‐MS) for further characterization.

### LC‐MS Analysis

Immunoprecipitated or total protein samples are either excised from SDS‐PAGE gels or directly subjected to trypsin digestion. The samples are first reduced with dithiothreitol (DTT) at 37 °C for 1 h, followed by alkylation with iodoacetamide (IAA) at room temperature in the dark for 30 min. Trypsin is then added at a 1:50 enzyme‐to‐protein ratio, and digestion is carried out overnight at 37 °C. The resulting peptides are extracted using an acetonitrile (ACN) and formic acid (FA) gradient, purified with C18 solid‐phase extraction (SPE) columns, and subsequently vacuum‐dried. The resulting peptide was analysed using a QExactive mass spectrometer coupled to a nano‐LC (AdvanceLC). The acquired spectra were analysed using the SEQUEST HT algorithm.

### Protein Interacting Analysis

Putative interacting proteins were identified after co‐IP and LC‐MS analysis in thp1 cells. The list of the potential interacting proteins was submitted to the STRING database (https://www.string‐db.org) and the protein interacting network was automatically obtained.

### T‐Cell Proliferation Assay

CD8^+^ T cells were obtained from donors’ peripheral blood using EasySep Direct Human CD8^+^ T cells Isolation Kit following the manufacture's protocol, T cells were labeled with CFSE and incubated with macrophages and cancer cells. T cells were then harvested and subjected to flowcytometry, the proliferation of T cells was analyzed.

### Proteomic Technology Phospho‐Proteomics Technology

Briefly, protein was extracted and digested, the tryptic peptides were subjected to mass spectrometer. The resulting MS/MS data were processed using Proteome Discoverer search engine (v.2.4). Tandem mass spectra were searched against Homo_sapiens_9606_SP_20230103.fasta (20389 entries) concatenated with reverse decoy and contaminants database. Trypsin (Full) was specified as cleavage enzyme allowing up to 2 missing cleavages. Min. peptide length was set as 6 and max. number of modification per peptide was set as 3. Mass error was set to 10 ppm for precursor ions and 0.02 Da for fragment ions. Carbamidomethyl on Cys was specified as fixed modification. Oxidation on Met, acetylation on protein N‐terminal, met‐loss on Met and met‐loss+acetyl on Met were specified as variable modification. False discovery rate (FDR) of protein, peptide and PSM was adjusted to <1%.

### Isolation of Tumor Infiltrated Macrophages

Under sterile conditions, freshly excised tumor tissues were collected and placed in a sterile culture dish containing calcium‐ and magnesium‐free phosphate‐buffered saline (PBS). The tissues were mechanically dissociated using a syringe plunger to maximize cell release. The minced tissues were then transferred to a 50 mL centrifuge tube, resuspended in PBS, and gently pipetted to facilitate dissociation. The resulting suspension was filtered through a 70 µm cell strainer to remove tissue debris and undigested extracellular matrix, yielding a single‐cell suspension. The suspension was centrifuged at 300 g for 5 min, the supernatant was discarded, and the cell pellet was resuspended in PBS. Tumor‐associated macrophages were enriched using the EasySep Direct Human Monocyte Isolation Kit (STEMCELL Technologies, Cat# 19669RF) via negative selection. According to the manufacturer's protocol, the single‐cell suspension was incubated with a cocktail of depletion antibodies, followed by the addition of magnetic particles for further incubation. The suspension was then placed in a magnet to capture non‐target cells, and the supernatant containing highly purified tumor‐associated macrophages was collected. The isolated macrophages were resuspended in ImmunoCult‐SF Macrophage Medium (STEMCELL Technologies, Cat# 10961) and seeded into sterile culture plates. Cells were maintained at 37 °C in a 5% CO₂ incubator, with media changes as required to sustain cell viability and functionality.

### Statistics and Reproducibility

Statistical analysis was carried out using Microsoft Excel 2019 (version 2503, build 16.0.18623.20116) and GraphPad Prism (version 5.0.0) for Windows. Experimental data are represented as the mean ± s.d. of a minimum of three biological replicates. Unless otherwise indicated, Student's two‐tailed unpaired t‐test was used to determine statistical significance of in vitro experiments. Gehan–Breslow–Wilcoxon test or log‐rank test was used to determine the statistical differences of the survival data. All statistical tests were two‐sided, and a *P* value of less than 0.05 was considered statistically significant. The following data were used: ribosome profiling, PRJCA022726, Proteome, IPX0011287000 at proteome X change.

## Conflict of Interest

The authors declare no conflict of interest.

## Supporting information



Supporting Information

Supporting Information

## Data Availability

The data that support the findings of this study are openly available in Ribosome profiling, PRJCA022726 at https://www.[url], reference number 22726.
