# Supplementary material for: LincNEAT1 Encoded‐NEAT1‐31 Promotes Phagocytosis by Directly Activating the Aurora‐A–PI3K–AKT Pathway
Source: Adv Sci (Weinh). 2025 May 8;12(30):2413473. doi: 10.1002/advs.202413473 (PMC12376509; doi:10.1002/advs.202413473)
Supplement: Supplementary file 1 — Supporting Information [file ADVS-12-2413473-s001.docx]

**
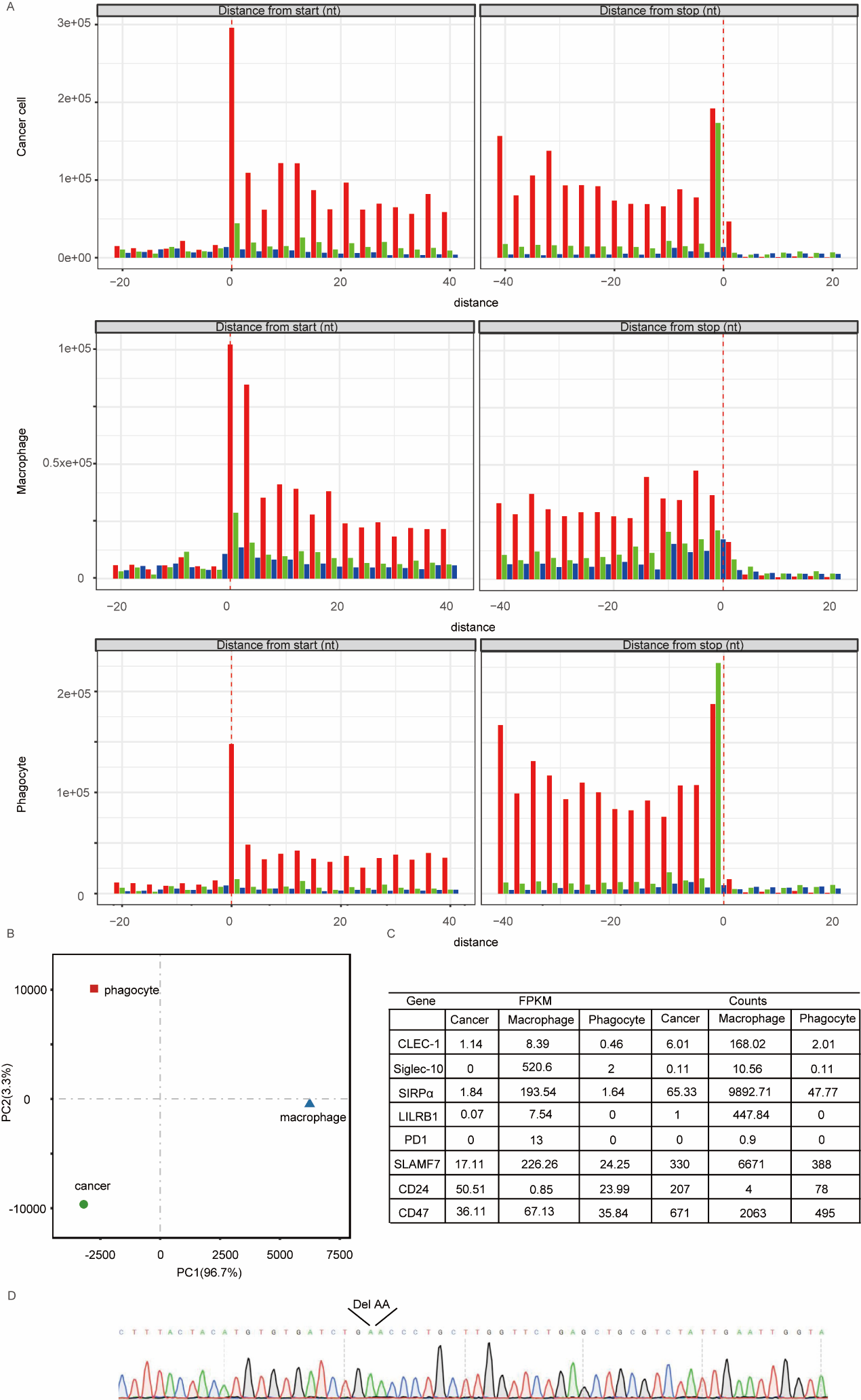
SFigure 1. Ribosome profiling of cancer cells, macrophages, and phagocytic macrophages are shown**

1. The reads of ribosome protect fragment mapped to the translation.
2. The principal component analysis of cancer cell, macrophage and phagocyte according to the RPF signature
3. The reads of indicated phagocytosis checkpoints in cancer cell, macrophage and phagocyte.
4. Sanger sequence of the CRISP-Case9 induced LincNEAT1 K.O. in HEK293T cells.

Abbreviations: RPFs= ribosome protect fragments; HEK= human embryonic kidney; CRISPR/Cas9= clustered regularly interspaced short palindromic repeats/CRISPR-associated protein 9; CD= cluster of differentiation; CLEC-1= C-type lectin-like receptor 1; Siglec-10= sialic acid-binding immunoglobulin-like lectin 10; SIRPα= signal regulatory protein alpha; LILRB1= leukocyte immunoglobulin-like receptor subfamily B1; PD1= programmed death1; SLAMF7= signaling lymphocytic activation molecule family 7; PCA= principal component analysis.


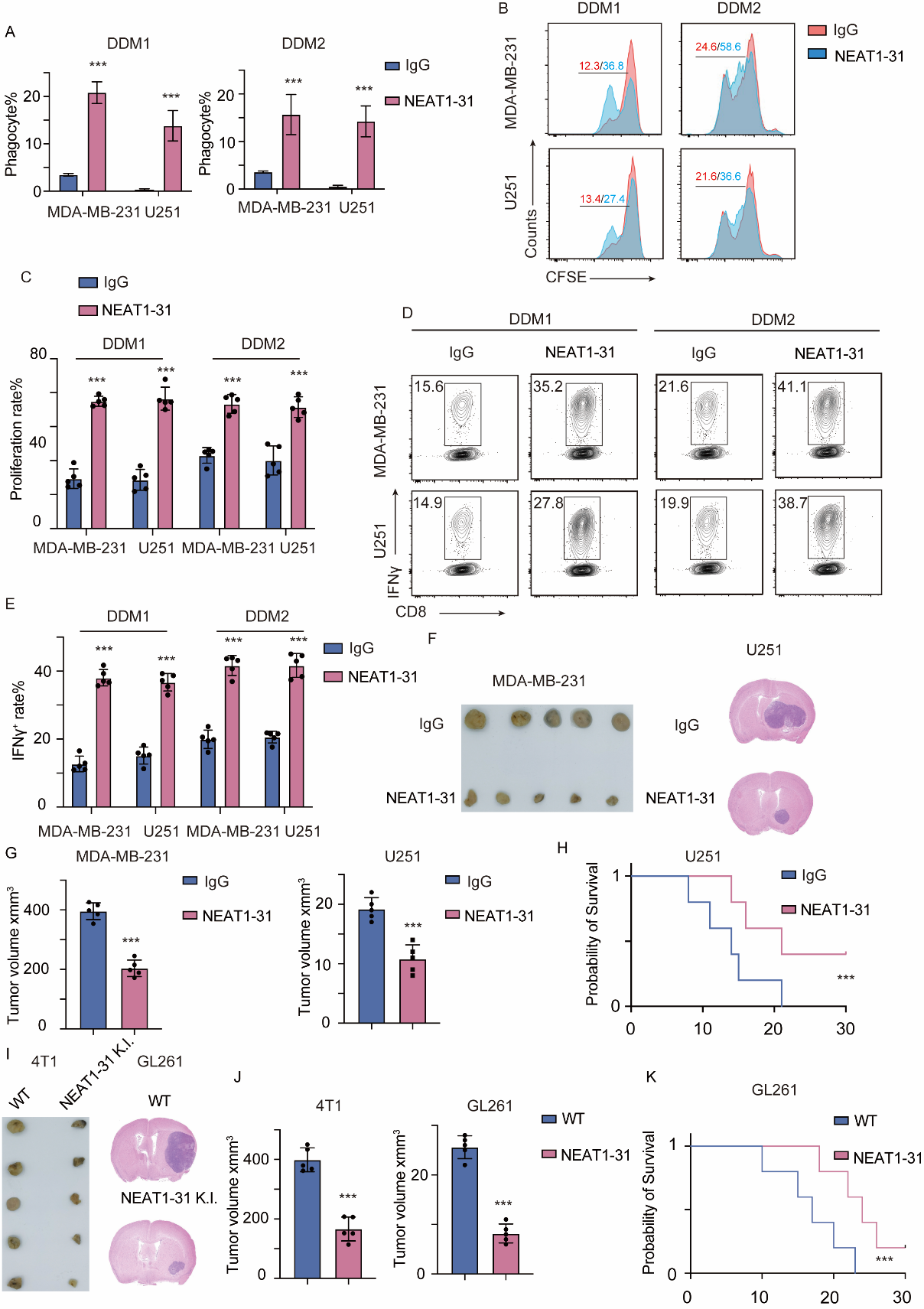


**SFigure 2. NEAT1-31 promotes phagocytosis against multi cancer cell-types**

1. The statistical analysis of phagocytosis against MDA-MB-231 and U251 of DDMs, n = 3, data was presented as mean±SD, unpaired T test, ***, p<0.001.
2. Human naïve T cells were sorted and co-culture with indicated cancer cells and DDMs, the proliferation of T cells was detected using T cell proliferation assay.
3. The statistical analysis of the proliferation rate of T cells with indicated modifications, n = 3, data was presented as mean±SD, unpaired T test, ***, p<0.001.
4. Cytometry detecting the IFNγ^+^ T cells described in B.
5. The statistical analysis of D, n = 3, data was presented as mean±SD, unpaired T test, ***, p<0.001.
6. The representative images of the tumors from mice bearing MDA-MB-231 and U251 with indicated modifications, n = 5 per group.
7. The statistical analysis of F, n = 5 per group, data was presented as mean±SD, unpaired T test, ***, p<0.001.
8. The overall survival time of mice bearing U251 is situ, n= 5 per group, log-rank test, ***, p<0.001.
9. The representative images of the tumors from mice bearing 4T1 and GL261 with indicated modifications, n = 5 per group, ***, p<0.001.
10. The statistical analysis of I, n = 5 per group, data was presented as mean±SD, unpaired T test, ***, p<0.001.
11. The overall survival time of mice bearing GL261 in situ, n= 5 per group, log-rank test, ***, p<0.001.

Abbreviations: NEAT= nuclear enriched abundant transcript; DDMs= donor-derived macrophages; IFNγ= interferon-γ.

All the experiments were repeated at least 3 times with similar results.


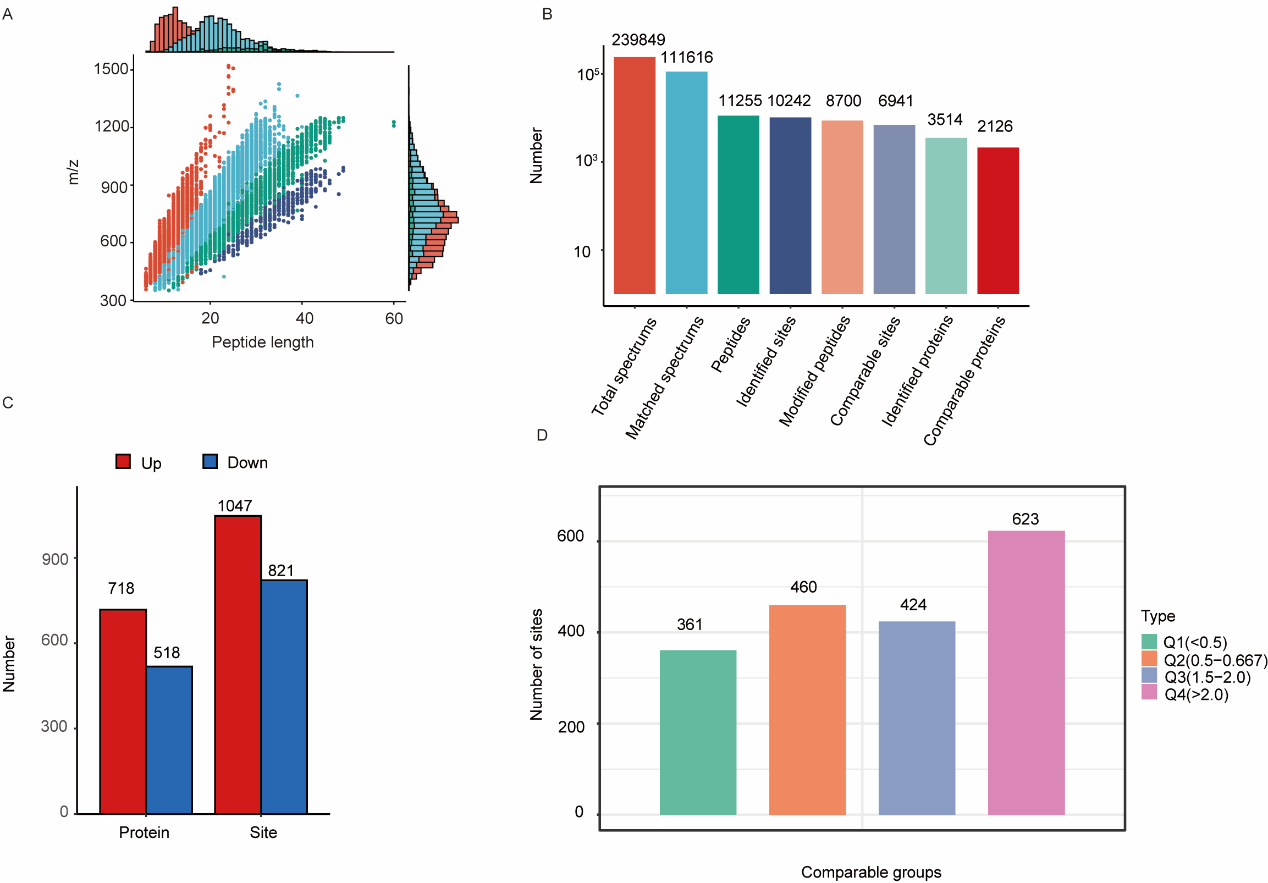


**SFigure 3. Phosphoproteomics analysis of NEAT1-31 K.O. cells**

1. The peptide length distribution and charge distribution.
2. The overall review of the phosphoproteomics analysis.
3. The details of dysregulated protein and dys-modified site.
4. The modified site in each goup.

Abbreviations: NEAT= nuclear enriched abundant transcript; K.O.=


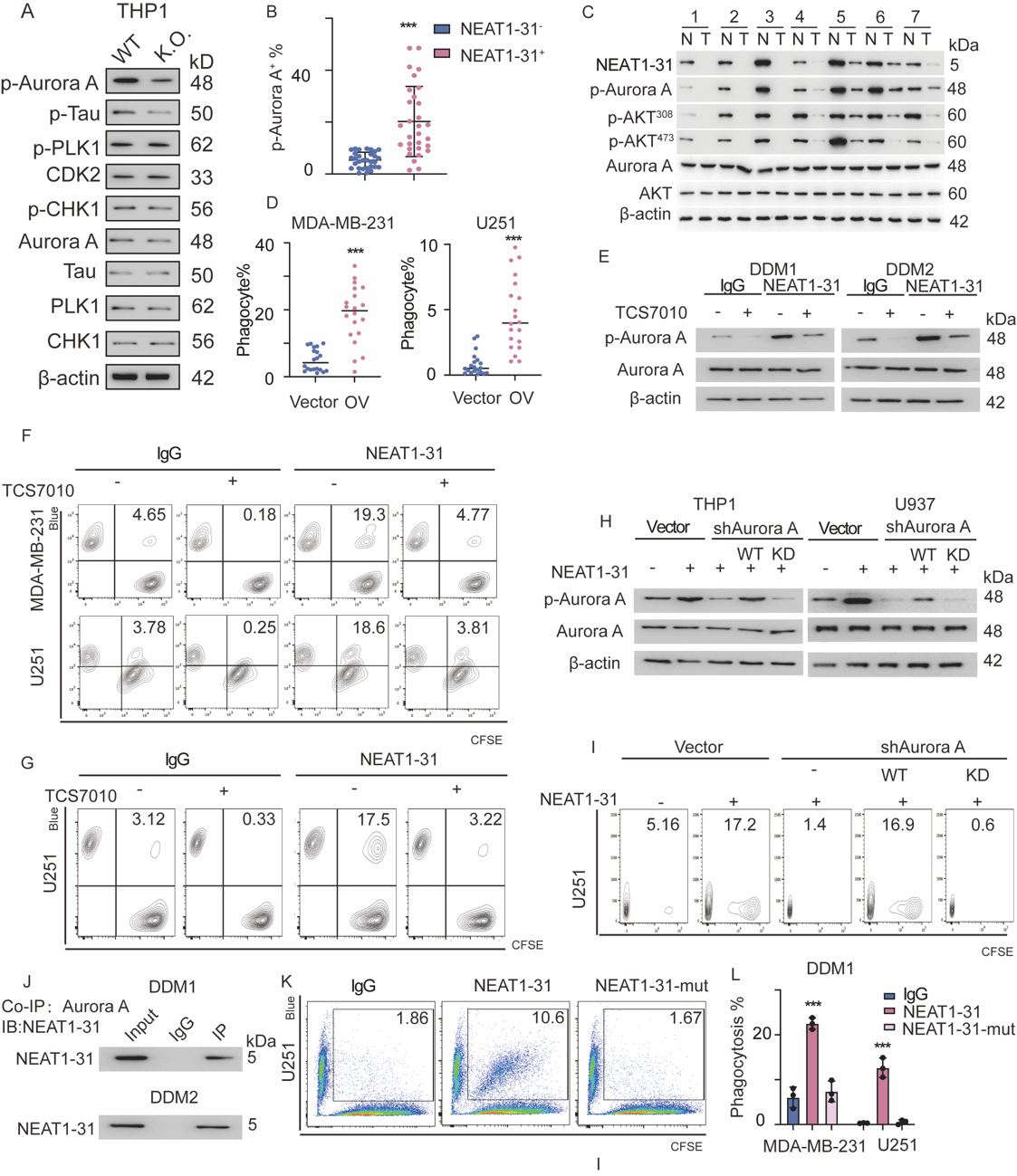


**SFigure 4. NEAT1-31-mediated Aurora-A activation promotes phagocytosis**

1. Immunoblot detecting p-Aurora A, p-Tau, p-PLK1, p-CHK1 in cells with indicated modifications.
2. Macrophages were divided into NEAT1-31 -/+ subgroups, the percentage of p-Aurora A^+^ was detected, data was presented as an individual dot, n = 40, paired T test, ***, p<0.001.
3. Macrophages were isolated from paired normal tissues and tumors, immunoblot was applied detecting p-Aurora A and AKT pathway.
4. THP1 was transfected with Aurora A plasmid, the phagocyte percentage was analyzed. data was presented as an individual dot, n = 20, paired T test, ***, p<0.001.
5. DDMs were treated with TCS7010 and subjected to immunoblot.
6. DDM1 were treated with TCS7010 and subjected to *in vitro* phagocytosis assay.
7. DDM2 were treated with TCS7010 and subjected to *in vitro* phagocytosis assay.
8. macrophages induced from U937/THP1 were transfected with small harpin RNA targeting Aurora A and re-express WT Aurora A and kinase dead (KD) Aurora A, p-Aurora A was detected using immunoblot.
9. *In vitro* phagocytosis of DDMs with indicated modifications.
10. Co-IP assay was applied using Aurora A antibody, the complex was subjected to immunoblot detecting NEAT1-31.
11. DDMs with indicated modifications were subjected to in vitro phagocytosis assay.
12. DDMs with indicated modifications were subjected to in vitro phagocytosis and the percentage of phagocyte was detected, n = 3, data was presented as mean±SD, Kruskal–Wallis test, Dunn’s post hoc test , ***, p<0.001.

Abbreviations: Ig= immunoglobulin; NEAT1= nuclear enriched abundant transcript; DDMs= donor-derived macrophages; KD= kinase-dead; THP-1= Tohoku Hospital Pediatrics-1; IB= immunoblotting; WT= wild-type.

All the experiments were repeated at least 3 times with similar results.


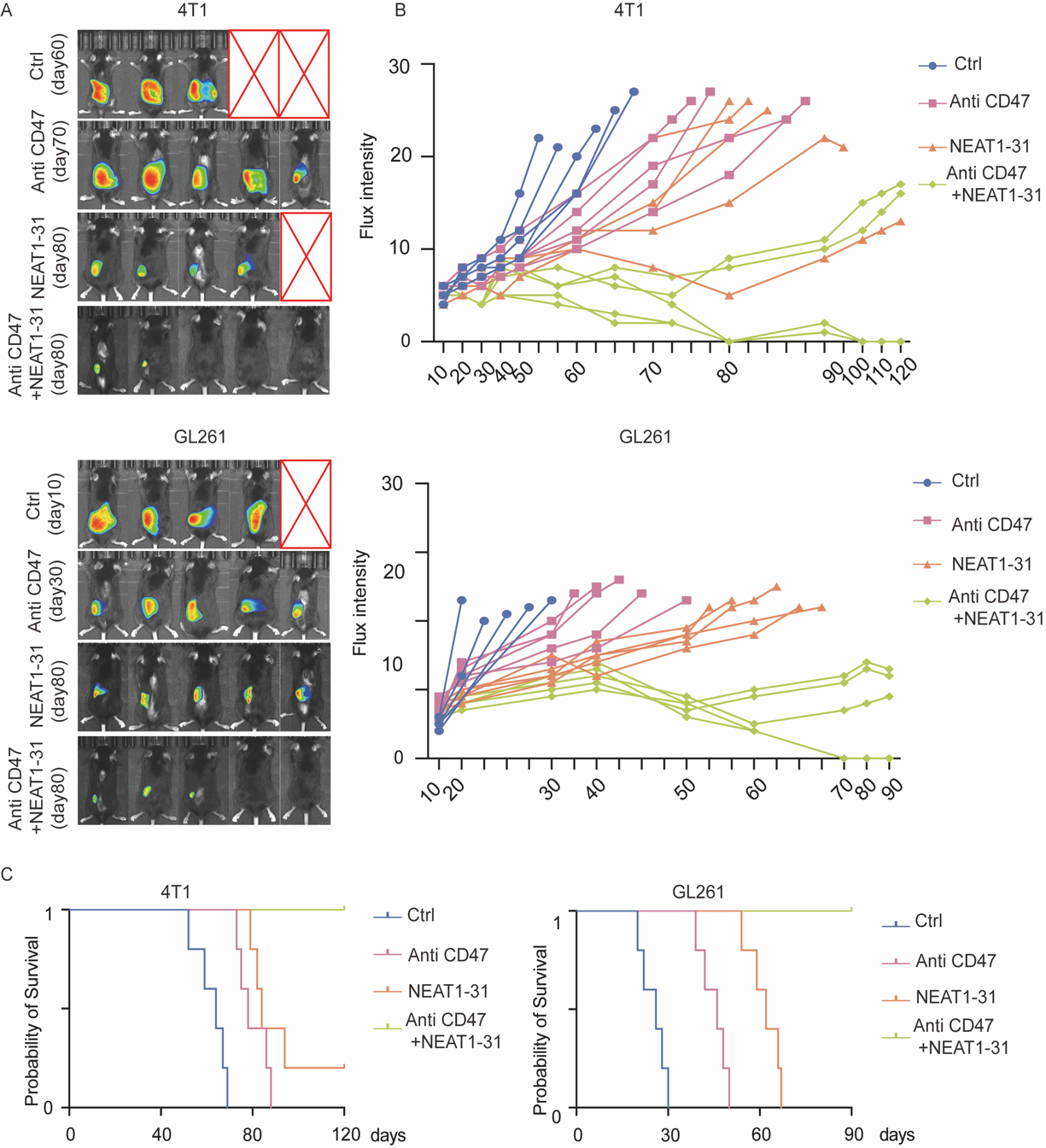


**SFigure 5. NEAT1-31 promotes anti-CD47 efficacy in C57 mice**

1. The representative images of flux intensity of mice bearing 4T1 and GL261 with indicated modifications, animal = 5 per group.
2. The statistical analysis of flux intensity in mice with indicated modifications, animal = 5 per group.
3. The overall survival analysis of mice bearing MDA-MB-231 and U251, animal = 5 per group.

All the experiments were repeated at least 3 times with similar results.

**Table 1: mRNA phagocytic macrophage activator**

**Table 2: LincRNA phagocytic macrophage activator**

**Table 3: 3‘UTR phagocytic macrophage activator**

**Table 4: 5’UTR phagocytic macrophage activator**
